# The Role of Hexokinase and Hexose Transporters in Preferential Use of Glucose over Fructose and Downstream Metabolic Pathways in the Yeast *Yarrowia lipolytica*

**DOI:** 10.3390/ijms22179282

**Published:** 2021-08-27

**Authors:** Piotr Hapeta, Patrycja Szczepańska, Tadeusz Witkowski, Jean-Marc Nicaud, Anne-Marie Crutz-Le Coq, Zbigniew Lazar

**Affiliations:** 1 Department of Biotechnology and Food Microbiology, Wrocław University of Environmental and Life Sciences, Chełmońskiego Street 37, 51-630 Wrocław, Poland; piotr.hapeta@upwr.edu.pl (P.H.); patrycja.szczepanska@upwr.edu.pl (P.S.); Tadeusz.Witkowski42@gmail.com (T.W.); 2 Université Paris-Saclay, INRAE, AgroParisTech, Micalis Institute, 78352 Jouy-en-Josas, France; jean-marc.nicaud@inrae.fr (J.-M.N.); anne-marie.le-coq@inrae.fr (A.-M.C.-L.C.)

**Keywords:** *Yarrowia lipolytica*, hexokinase, hexose transporters, glucose, fructose, erythritol

## Abstract

The development of efficient bioprocesses requires inexpensive and renewable substrates. Molasses, a by-product of the sugar industry, contains mostly sucrose, a disaccharide composed of glucose and fructose, both easily absorbed by microorganisms. *Yarrowia lipolytica*, a platform for the production of various chemicals, can be engineered for sucrose utilization by heterologous invertase expression, yet the problem of preferential use of glucose over fructose remains, as fructose consumption begins only after glucose depletion what significantly extends the bioprocesses. We investigated the role of hexose transporters and hexokinase (native and fructophilic) in this preference. Analysis of growth profiles and kinetics of monosaccharide utilization has proven that the glucose preference in *Y. lipolytica* depends primarily on the affinity of native hexokinase for glucose. Interestingly, combined overexpression of either hexokinase with hexose transporters significantly accelerated citric acid biosynthesis and enhanced pentose phosphate pathway leading to secretion of polyols (31.5 g/L vs. no polyols in the control strain). So far, polyol biosynthesis was efficient in glycerol-containing media. Moreover, overexpression of fructophilic hexokinase in combination with hexose transporters not only shortened this process to 48 h (84 h for the medium with glycerol) but also allowed to obtain 23% more polyols (40 g/L) compared to the glycerol medium (32.5 g/L).

## 1. Introduction

The oleaginous yeast *Yarrowia lipolytica* is an industrial micro-organism used for production of complex biological compounds such as tailor-made lipids [1,2], biofuel precursors [3] or industrial and therapeutic enzymes [4,5], as well as more compact molecules such as acidifying agent citric acid [6] and low-calorie sweeteners, including erythritol and mannitol [7,8]. Substrates can be chosen among hydrophobic carbon sources (*n*-alkanes, oils and fats) and hydrophilic ones, such as glycerol, monosaccharides and organic acids. From the latter, glycerol is a superior carbon source for *Y. lipolytica*, due to its rapid and preferred use over glucose when supplied in a mixture [9,10,11]. To date, production of erythritol by this yeast relies on the use of glycerol in the culture medium as it acts as a stress factor, increasing osmotic pressure required for erythritol overproduction [12]. In turn, the narrow spectrum of monosaccharides used by wild-type *Y. lipolytica* strains, which includes glucose, fructose and mannose [13], can be extended by genetic engineering to galactose and xylose [14,15,16]. Glucose and fructose are readily available substrates present in plant biomass and industrial by-products such as molasses [17]. Many yeast species, including *Saccharomyces cerevisiae*, show a preferential uptake of glucose over fructose in mixed sugar media [18]. This trait is particularly prominent in *Y. lipolytica*, for which several experiments enlightened that fructose uptake begins only after glucose is completely or mostly depleted from the medium [19,20]; the molecular mechanism behind remains unknown. In order to enter the metabolism, monosaccharides need first to be taken up by the cell, a step which requires transporters, then to be activated through phosphorylation by hexose kinases.

*Y. lipolytica* possesses six *bona fide* hexose transporters (encoded by the genes *YHT1* to *YHT6*) belonging to the 24-protein Sugar Porter (SP) family [20]. Among them, Yht1, Yht3 and Yht4 are efficient broad-range hexose transporters enabling glucose, fructose, mannose and galactose uptake as determined by the heterologous complementation study [20]. Yht1 and Yht4 were identified as the main active hexose transporters in this yeast as deletion of both genes prevented growth on the four hexoses [20]. Furthermore, out of the six *YHT* genes, only *YHT1* (*YALI0C06424g*) and *YHT4* (*YALI0E23287g*) were consistently transcribed during the growth of W29 and H222 strains in glucose- and fructose-based media [20]. These were also the only Yht transporters expressed in media containing glucose, glycerol and a mixture of both under nitrogen-limiting conditions, showing a differential expression pattern depending on carbon source and nitrogen concentration [9]. *YHT3* (*YALI0F19184g*) appeared to be poorly transcribed in the conditions tested. Moreover, the strain-dependent polymorphism in this gene had an impact on the efficiency of the transporter as the gene in *Y. lipolytica* H222 encoded a much more efficient transporter than its counterpart from the W29 strain [20].

Two enzymes with hexokinase activity have been identified in *Y. lipolytica*. One of them is a glucokinase, with glucose as the only substrate; the other one phosphorylates glucose, fructose and mannose [21] and is encoded by the *YlHXK1* (*YALI0B22308g*) gene. The primary structure of YlHxk1 has quaint characteristics absent in other known hexokinases. In addition to conserved domains allowing for the action on hexoses, YlHxk1 contains an atypical 37-amino acid loop spanning between glucose- and ATP-binding domains [21], which indirectly influences enzyme activity [22]. The overexpression of *YlHXK1* enhanced fructose utilization in the *Y. lipolytica* strains showing tedious growth on this monosaccharide, such as the wild strain W29, and more generally improved hexose utilization, increased carbon flux through glycolysis, changed the preferential use of glycerol to glucose when both substrates were present and reduced filamentation [20,22,23]. It also boosted lipid biosynthesis from fructose in an obese *Y. lipolytica* strain up to 55% [24]. The overexpression had, however, no effect on preferential uptake of glucose over fructose. The preference might in turn result from the kinetic properties of YlHxk1 as the enzyme has higher affinity for glucose than for fructose with a Km of 0.38 mM and 3.56 mM, respectively [21], even more pronounced than for the ScHxk2 hexokinase of *S. cerevisiae* (0.25 mM for glucose; 1.5 mM for fructose). In contrast, hexokinase from *Schizosaccharomyces pombe* (SpHxk1) is fructophilic with a Km of 8.4 mM for glucose and 1.5 mM for fructose [25].

In this study we investigated the role of hexose transporters and hexokinase in the preferential uptake of glucose over fructose in *Y. lipolytica*. We first examined the kinetic properties of Yht1, Yht3 and Yht4 in a heterologous host and then, through overexpression of hexose transporters in combination with native YlHxk1 or heterologous SpHxk1, we showed rearrangement of glucose and fructose utilization profiles. We further indicated that the increased carbon flux can be directed to different metabolic pathways by changing cultivation conditions. In addition, we propose that the introduced modifications can be harnessed for production of polyols by *Y. lipolytica* grown in hexose-containing media.

## 2. Results

### 2.1. Expression of Y. lipolytica Hexose Transporters in Heterologous Host Induce Diverse Monosaccharide Utilization Phenotypes

The heterologous host *S. cerevisiae* EBY.VW4000 devoid of hexose transporters [26] is well-suited for comparing hexose uptake characteristics conferred by single transporters and has been previously used for this purpose [20,27,28]. The *S. cerevisiae* strains expressing *YHT1*, *YHT3*_H222_ (from *Y. lipolytica* H222, named hereafter *YHT3*) or *YHT4* were cultivated in media containing glucose, fructose, mannose and a mixture of glucose and fructose. To monitor the consumption of each monosaccharide, substrate concentrations were measured throughout the 48 hgrowth. 

**Figure 1:**
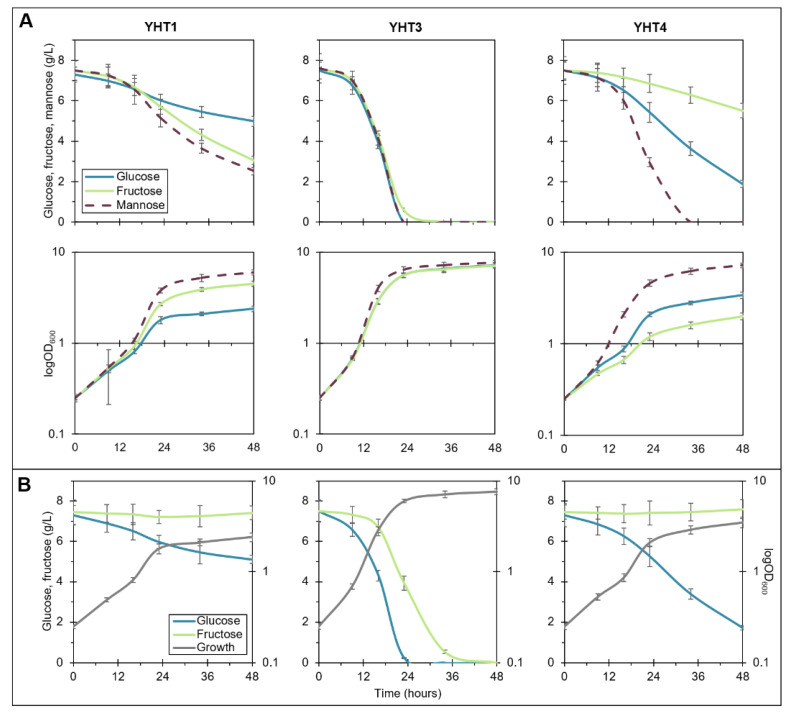
Sugar utilization and growth of *S. cerevisiae* EBY.VW4000 transformants expressing hexose transporters from *Y. lipolytica* in single sugar media (**A**) and medium containing a mixture of glucose and fructose (**B**). The cultivations were carried out in three biological replicates.

Single sugar experiments pointed out different efficiencies for sugar uptake among the three transporters (Figure 1A). During the 48 h of cultivation, only the strain expressing *YHT3* completely consumed each substrate. This strain grew slightly better on mannose than on glucose and fructose. In turn, *S. cerevisiae* with *YHT1* utilized fructose more efficiently, while the YHT4 strain exhibited a clear preference towards glucose. Both of the strains, however, preferred mannose over glucose and fructose, and these differences were also visible in the growth profiles. Strikingly, the *YHT1*-expressing strain was unable to completely utilize any of the available substrates within 48 h. 

When grown in a medium containing a mixture of glucose and fructose, all the strains showed a clear glucose preference (Figure 1B). Only the *YHT3*-expressing strain completely utilized both sugars within the given timeframe. In contrast, the *YHT1*- and *YHT4*-expressing strains did not consume fructose at all, and the YHT4 strain used glucose at a 2.6-fold higher rate than the YHT1 strain.

### 2.2. Construction and Growth Profiling of Y. lipolytica Strains Overexpressing Hexose Transporters and Hexokinases

The different characteristics of sugar uptake observed in *S. cerevisiae* EBY.VW4000 with Yht1, Yht3 and Yht4 encouraged us to examine the behavior of *Y. lipolytica* expressing them individually and in combinations. Previously engineered *yht1-4Δ* mutant of *Y. lipolytica* W29 (JMY4788) impeded in hexose transport was used as the parental strain. Transporters were associated with two alternative hexokinases: the native YlHxk1 and SpHxk1 from *S. pombe* known for its higher affinity for fructose. First, expression cassettes containing the pTEF-driven *YlHXK1* and *SpHXK1* genes were introduced into *YHT1-4Δ* mutant. From these two strains, derivatives with the desired combinations of transporters were developed. The *YHT* genes were placed under control of strong, constitutive pTEF promoter (see Section 4 for details). In order to avoid artefacts caused by random integration of the cassettes in the genome, three clones for each genotype were analyzed for their growth ability in media containing glucose, fructose and mixture of both sugars. In the microcultivation experiments the transformants of the same genotype exhibited similar growth profiles, presented as average growth curves in Figure S1. All strains were able to grow in glucose and/or fructose, however, with varying rates. Thus, *YHT4* caused poor growth in fructose when associated with *SpHXK1* in the strains SpH1-Y4 and SpH1-Y1-Y4 (Figure S1D). Major variations were observed with *YHT1*, which conferred slow growth in fructose, in association with either hexokinase. Co-expression of both *YHT1* and *YHT4* in the SpH1 strain enabled weak growth in fructose (Figure S1). Only subtle differences were observed for the remaining transformants. 

### 2.3. Rearrangement of Sugar Utilization Profile through Overexpression of Hexose Transporters in Combination with Hexokinases in Y. lipolytica

To analyze their sugar preference, transformants were further characterized for their substrate consumption profiles during their growth in flasks containing a minimal medium supplemented with glucose and fructose as a mixture. Two strains of each genotype were used in the following experiment. Each genotype conferred the same phenotypic changes; therefore, data for one strain of each genotype is presented. 

**Figure 2:**
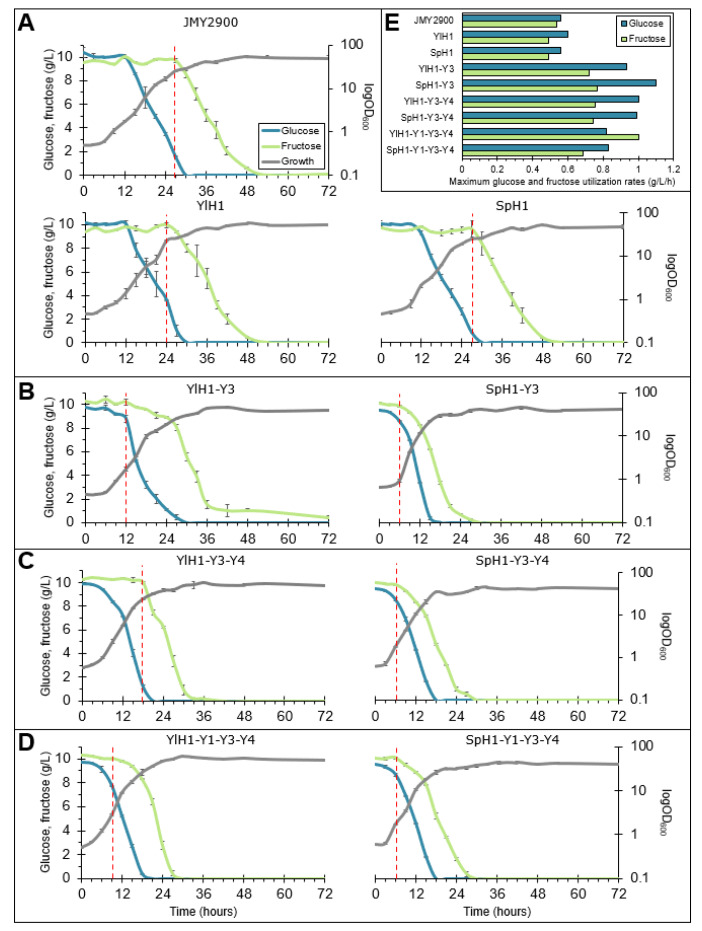
Growth and substrate utilization profiles of the *Y. lipolytica* transformants expressing combinations of hexokinases and hexose transporters in flasks in a defined medium containing a mixture of glucose and fructose (**A**–**D**). Maximum glucose and fructose utilization rates (**E**). Red vertical dashed lines indicate onset of fructose utilization. The cultivations were performed in three biological replicates.

All of the constructed strains used glucose prior to fructose; however, differences in the consumption rates of individual hexoses were observed. In the background of wild-type transporter genes at their original loci, the native *YlHXK1* (control strain JMY2900) as well as the additional pTEF-*YlHXK1* and pTEF-*SpHXK1* cassettes resulted in similar consumption profiles (Figure 2A); glucose was depleted around 30 h of culture, fructose uptake started at about 24–27 h and was completed at around 50 h. Overexpressing the transporter genes, in any combination along with the hexokinases, accelerated hexose consumption, leading to glucose and fructose depletion within 15–21 h and 27–33 h, respectively. One striking exception was observed with the YHT3 transporter, which the effect of strongly varied depending on the hexokinase. Overexpression of both *YHT3* and *YlHXK1* resulted in an early onset of fructose utilization, at around 12 h of culture (Figure 2B). Despite that, the initial consumption rate was inconsiderable and increased only when glucose was completely exhausted and ceased at around 36 h. In contrast, the co-expression of *SpHXK1* and *YHT3* greatly improved utilization of both glucose and fructose. The sugars were consumed nearly in parallel from as early as 6 h of culture and were completely depleted within 30 h. This result places the SpH1-Y3 strain among the best ones for hexose uptake. The difference between both strains was also clear in their growth profiles, as the YlH1-Y3 mutant showed slower growth, reaching stationary phase 18 h later than its *SpHXK1*-expressing counterpart. Interestingly, when either *YHT1* or *YHT4* were expressed with *YlHXK1* and *YHT3*, the growth and substrate utilization improved (Figure 2C). The same transporter complementation in the *SpHXK1–YHT3* background did not much modify its phenotype. On the other hand, expression of the three transporters with either of the hexokinases resulted in a similar phenotype, close to that observed for other strains expressing *SpHXK1* and *YHT3* (Figure 2D). Interestingly, the maximum glucose utilization rates were decreased in the strains expressing three transporters (Figure 2E). Furthermore, fructose was consumed 1.4-fold faster by the YlH1-Y1-Y3-Y4 strain than by the *SpHXK1*-expressing mutant with the same set of transporters, while glucose was consumed at the same rate. The remaining eight strains did not exhibit distinct differences in terms of growth and substrate utilization (Figure S2).

In sum, the identity of both transporter and hexokinase, had strong impact on sugar utilization profiles. All the strains with *SpHXK1* and *YHT3* overexpressed and only them started to consume fructose very early, in the time when glucose had hardly begun to be consumed. In the following step, we were interested to examine the behavior of the strains in the strictly controlled bioreactor conditions. In particular, we wondered whether the rapid and parallel substrate utilization by the strains expressing *SpHXK1* and *YHT3* could outperform the other strains in a biotechnological process and lead to its shortening. For this purpose, we chose the process of citric acid (CA) production, well-described for *Y. lipolytica*. 

### 2.4. The Input Flux of Hexoses Impacts Downstream Metabolic Pathways

**Figure 3:**
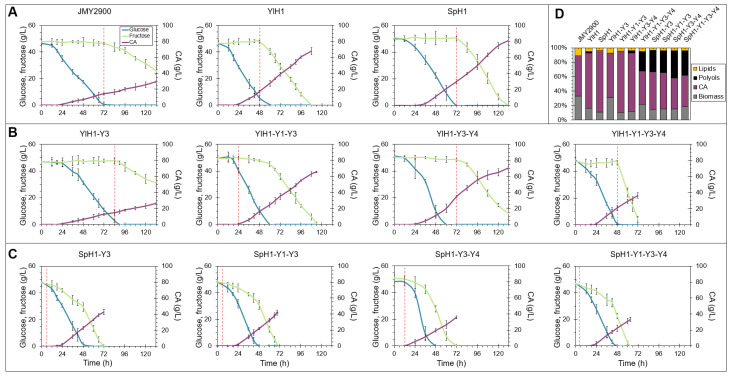
Sugar consumption and citric acid (CA) secretion of *Y. lipolytica* strains grown in a media optimized for citric acid production in 5 L stirred tank bioreactors (**A**–**C**); metabolic profiles of the analyzed strains (**D**). Red vertical dashed lines indicate the onset of fructose utilization. The cultivations were performed in three biological replicates.

For strain comparisons in the process of CA production, the cultivations were carried out in a defined medium optimized for CA biosynthesis containing a mixture of glucose and fructose under carbon excess and nitrogen limitation in 5 L stirred-tank bioreactors. In general, sugar consumption profiles were similar to that described in the previous section. The rates of CA production and final titers depended greatly on HXK/YHT genotypes (Table S2). The 17 bioreactor fermentations (one strain of each genotype) were performed in triplicates and the results are summarized in supplementary Figure S3. The major phenotypes are depicted in Figure 3 and discussed below. The cultures were carried out until both substrates were completely utilized or until 132 h in the case of the JMY2900, YlH1-Y3 and YlH1-Y3-Y4 strains.

Overexpression of either hexokinase in the native *YHT* background accelerated consumption of both hexoses and increased CA production 2.3- (*p* = 6.2 × 10^−6^) and 2.7-fold (*p* = 1.0 × 10^−6^; 67.9 g/L and 81.1 g/L vs. 29.4 g/L) for the YlH1 and SpH1 strain, respectively (Figure 3A). These two strains showed the highest CA yields from glucose and fructose among the analyzed transformants (Table 1). A 24 h earlier onset of fructose utilization was observed for the YlH1 strain. The control JMY2900 strain was unable to consume all the available fructose within 132 h of culture. In turn, *YHT3* in combination with *YlHXK1* had a detrimental effect on sugar consumption and CA biosynthesis when compared to the YlH1 with native transporters (Figure 3B). Conversely, the expression of an additional transporter (YHT1) restored the phenotype of the YlH1 strain, while the introduction of YHT4 resulted in an intermediate phenotype of the parental and *YlHXK1*-expressing strains. When three transporters were overexpressed alongside *YlHXK1*, sugars were completely exhausted within 72 h, but the CA levels showed a two-fold decrease when compared to the mutants with two transporters (Figure 3B). Nevertheless, due to improved sugar consumption characteristics, the CA productivities were largely comparable to that of the YlH1 strain (Table 1).

*YHT3* combined with *SpHXK1* clearly accelerated utilization rates of glucose and fructose and this effect was consistent regardless of additional transporters (Figure 3C). The strain SpH1-Y3 as well as SpH1-Y1-Y3 showed similar, yet noticeably, decreased CA secretion when compared to the SpH1 strain. Transformants expressing SpHxk1 in combination with *YHT3* and *YHT4*, only *YHT4* or all three transporters had even lower CA biosynthetic abilities (Figure 3C and Figure S3). The obtained data showed that the observed decrease was accompanied by an increased polyol secretion of which mannitol and erythritol were predominant (Figure 3D, Table 1).

### 2.5. Overexpression of a Fructophilic Hexokinase and Three Hexose Transporters Induces Rapid Biosynthesis of Polyols from Glucose and Fructose

**Figure 4:**
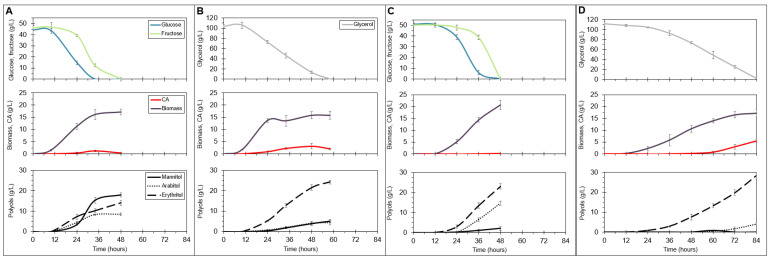
Substrate utilization, growth, citric acid (CA) and polyol production by the strain *Y. lipolytica* SpH1-Y1-Y3-Y4 in 5 L stirred-tank bioreactors in media optimized for polyol production containing a mixture of glucose and fructose (**A**) or glycerol (**B**); in media optimized for erythritol production containing a mixture of glucose and fructose (**C**) or glycerol (**D**). The cultivations were performed in three biological replicates.

Significant amounts of polyols produced by the *Y. lipolytica* strains overexpressing *SpHXK1* and *YHT3* in various combinations held promise that their secretion might be further enhanced by optimizing culture conditions. To verify this, the strain SpH1-Y1-Y3-Y4 was chosen as it showed good polyol production accompanied by nearly parallel consumption of glucose and fructose and the fastest fermentation time (Figure 3C). The mutant was cultivated in a media optimized for polyol biosynthesis containing either a mixture of glucose and fructose or glycerol as a carbon source (Figure 4). In the sugar-based medium, the substrates were rapidly utilized and completely exhausted within 48 h. The strain secreted 25-fold less CA than in the CA-optimized media but instead 1.5-fold more polyols (25.96 g/L vs. 40.58 g/L) of which 44% mannitol, 35% erythritol and 21% arabitol (Table 2 and Table S3). Mannitol production accelerated significantly with the onset of linear fructose consumption and lasted between 24 and 34 h of culture (Figure 4A). In turn, when glycerol was used, a different quantitative polyol spectrum was obtained in which erythritol was predominant (Figure 4B). Low amounts of mannitol, arabitol and CA were secreted while the biomass was comparable to that in the sugar-based medium. Glycerol was used within 58 h.

The SpH1-Y1-Y3-Y4 mutant was additionally tested in the media containing NaCl which was applied in order to increase osmotic pressure and promote erythritol biosynthesis. In the glucose- and fructose-based media the strain consumed available sugars within 48 h and produced 23.12 g/L erythritol, 14.66 g/L arabitol, negligible amounts of mannitol and no CA throughout (Figure 4C). In turn, when grown on glycerol, the transformant needed 36 h more to entirely utilize the substrate. Glycerol clearly favored erythritol biosynthesis at an expense of decreased arabitol production; however, in the final stage of the process, CA secretion occurred (Figure 4D). Compared to the cultivation on monosaccharides, mannitol was not detected and nearly 20% less biomass was made (Table 2). Interestingly, maximum glucose and fructose utilization rates were, respectively, 1.1- and 1.3-fold higher in the erythritol medium than in the polyol biosynthesis broth. Conversely, glycerol was consumed with a 2.1-fold slower rate in the erythritol medium when compared to the polyol medium. 

## 3. Discussion

In the *Y. lipolytica* genome, there are six genes encoding hexose transporters (*YHT1* to *YHT6*) of which *YHT1*, *YHT3* and *YHT4* code for efficient broad-range transporters enabling uptake of glucose, fructose, mannose and galactose [20]. The *YHT1* and *YHT4* genes were previously shown to be consistently transcribed in media containing glucose and fructose, and are, therefore, deemed the main hexose transporter-encoding genes [20]. On the other hand, *YHT3* expression was observed mostly in the stationary phase and occasionally in the exponential growth phase [20], suggesting an auxiliary role of this transporter in sugar uptake. In our study, we used previously constructed *S. cerevisiae* strains expressing YHT1, YHT3 (*YHT3*_H222_ allele from *Y. lipolytica* H222) and YHT4 [20], based on the EBY.VW4000 strain devoid of all 17 hexose transporters [26] to determine the kinetic characteristics of each transporter. To our surprise, YHT3 turned out to be the most efficient one when expressed in a heterologous host, enabling rapid utilization of glucose, fructose and mannose. It is, therefore, interesting that its expression was previously observed mainly in the stationary growth phase in *Y. lipolytica* [20]. Furthermore, all three transporters showed clear preference towards mannose which is not without significance for future studies on utilization of mannose-containing lignocellulosic biomass in bioprocesses using *Y. lipolytica*. 

**Figure 5:**
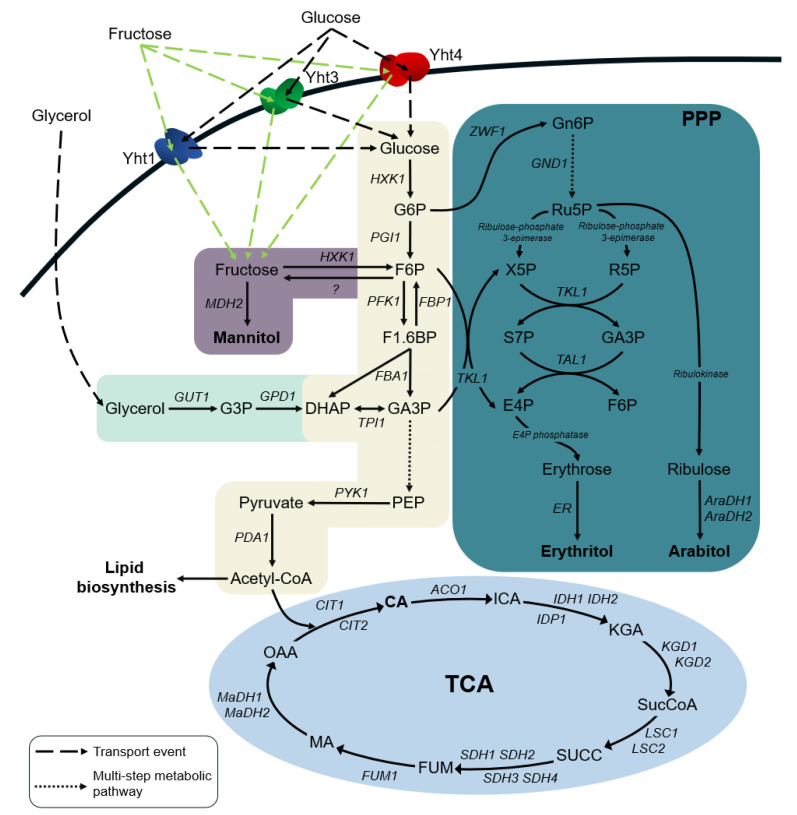
Simplified schematic representation of the metabolic pathways for polyol and CA biosynthesis in the yeast *Y. lipolytica*. Abbreviations: CA—citric acid; DHAP—dihydroxyacetone phosphate; E4P—erythrose 4-phosphate; F1.6BP—fructose 1.6-bisphosphate; F6P—fructose 6-phosphate; FUM—fumaric acid; G3P—glycerol 3-phosphate; G6P—glucose 6-phosphate; GA3P—glyceraldehyde 3-phosphate; Gn6P—gluconolactone 6-phosphate; ICA—isocitric acid; KGA—α-ketoglutarate; MA—malic acid; OAA—oxaloacetate; PEP—phosphoenolpyruvate; PPP—pentose phosphate pathway; R5P—ribose 5-phosphate; Ru5P—ribulose 5-phosphate; S7P—sedoheptulose 7-phosphate; SUCC—succinic acid; SucCoA—succinyl-CoA; TCA—tricarboxylic acid cycle; X5P—xylulose 5-phosphate. Gene identifiers: *ACO1 (YALI0D09361g)*; *AraDH1 (YALI0F02211g)*; *AraDH2 (YALI0E05643g)*; *CIT1 (YALI0E00638g)*; *CIT2 (YALI0E02684g)*; *ER (YALI0F18590g)*; *FBA1 (YALI0E26004g)*; *FBP1 (YALI0A15972g)*; *FUM1 (YALI0C06776g)*; *GND1 (YALI0B15598g)*; *GPD1 (YALI0B02948g)*; *GUT1 (YALI0F00484g)*; *HXK1 (YALI0B22308g)*; *IDH1 (YALI0E05137g)*; *IDH2 (YALI0D06303g)*; *IDP1 (YALI0F04095g)*; *KGD1 (YALI0E33517g)*; *KGD2 (YALI0E16929g)*; *LSC1 (YALI0E24013g)*; *LSC2 (YALI0D04741g)*; *MaDH1 (YALI0D16753g)*; *MaDH2 (YALI0E14190g)*; *MDH2 (YALI0D18964g)*; *PDA1 (YALI0F20702g)*; *PFK1 (YALI0D16357g)*; *PGI1 (YALI0F07711g)*; *PYK1 (YALI0F09185g)*; *SDH1 (YALI0D11374g)*; *SDH2 (YALI0D23397g)*; *SDH3 (YALI0E29667g)*; *SDH4 (YALI0A14784g)*; *TAL1 (YALI0F15587g)*; *TKL1 (YALI0E06479g)*; *TPI1 (YALI0F05214g)*; *ZWF1 (YALI0E22649g)*.

Microbial growth from a carbon source results from both transport and downstream metabolic pathways for energy and metabolite production. As previously reported, phosphorylation of fructose by hexokinase for entering glycolysis (Figure 5) is a limiting step in the *Y. lipolytica* wild-type W29 strain [24]. In order to examine the effect of individual transporters on hexose utilization by *Y. lipolytica*, we used the *YHT1-4Δ* mutant of W29 (strain JMY4788) impeded in hexose transport, unable to grow on glucose- nor on fructose-containing solid media (Figure S4). We previously constructed an overexpression cassette by placing the native hexokinase *YlHXK1* gene under the strong and constitutive pTEF promoter (pTEF-*YlHXK1*) [24]. As some hexokinases exhibit higher affinity towards fructose than for glucose, such as the SpHxk1 [25], an expression cassette with the pTEF-driven *SpHXK1* was constructed. We generated strains bearing either the *YlHXK1* or the *SpHXK1* overexpression cassette in the wild-type derivative strain (JMY2101) and in the auxotrophic *YHT1-4Δ* mutant derivative (PHY130) additionally overexpressing each of the three essential transporters, namely YHT1, YHT3_H222_ and YHT4 individually, in combinations of two and in a combination of three transporters. 

Previous experiments showed that wild-type *Y. lipolytica* strains grow poorly on fructose, but this can be overcome by hexokinase overexpression [24]. The introduction of heterologous hexokinase exerted the same effect as evidenced here with SpHxk1 or previously with ScHxk2 and YayaHxk1 [22]. The interplay between hexokinase and hexose transporters, however, seem to be crucial in the monosaccharide metabolism, especially fructose, in *Y. lipolytica* as clear differences in growth profiles, depending on the particular transporters associated with either hexokinase, were observed. The true nature of these interactions cannot be inferred using only growth profiles. We hypothesized that the molecular mechanism controlling preferential uptake of glucose over fructose in *Y. lipolytica* might hold in the affinity toward glucose and fructose of hexose transporters and/or hexokinase. YlHxk1 has over 9-fold higher affinity for glucose than for fructose [21], which could be one of the reasons for the yeasts’ strict glucose preference. As mentioned above, some yeast species, such as *S. pombe,* have fructophilic hexokinase with a higher affinity for fructose than for glucose [25]. In our study, overexpression of SpHxk1 did not change the substrate utilization pattern as glucose was always utilized first. This, however, might have occurred due to the existence of an intact glucokinase that could suffice for efficient glucose processing. 

The interplay between hexokinase and sugar transporter(s) was relevant, as, for example, YHT3 exerted varying effects on fructose utilization depending on the co-expressed hexokinase. Thus, when combined with YlHxk1, the onset of fructose consumption took place only after glucose depletion. Furthermore, fructose was imported to the cells until its concentration reached a threshold concentration of ca. 1.5 g/L in the shake-flask experiment, after which it was consumed with a very slow rate. In turn, YHT3 co-expressed with SpHxk1 triggered an entirely different phenotype allowing for rapid, nearly parallel utilization of both sugars to their complete exhaustion. In the SpHxk1 strains, the strong effect of YHT3 was present, while in the YlHxk1 background, additional transporters allowed for complete utilization of fructose, but in none of them parallel consumption of sugars was observed. The polarized, hexokinase-dependent effect of YHT3 suggests the interplay between YHT3 and hexokinase. It may be possible that the native Hxk1, which has a low affinity for fructose, directly or indirectly affects fructose transport through YHT3. On the other hand, the low Km for fructose of SpHxk1 allows for rapid fructose conversion. Furthermore, SpHxk1 might not interact with native proteins in the same manner as YlHxk1 does, therefore, allowing for uninterrupted fructose import. Structurally, YlHxk1 has a unique feature, namely a 37-amino acid loop not found in other known hexokinases, including SpHxk1 (Figure S5; [21]), which has been shown to be important in inhibitor binding and gene expression regulation [22]. This structure could, therefore, interact with YHT3 in effect decelerating sugar intake and defending the cell from the carbon overflow in the central carbon metabolism (Figure 5) and/or increased intracellular osmotic pressure.

The efficiency of substrate(s) utilization remains an important aspect in bioprocess design. Considering the rapid consumption of glucose and fructose by the *SpHXK1* and *YHT3*-expressing strains, we were interested to see how they would perform in a biotechnological process. As a model bioprocess, we chose the extensively studied in *Y. lipolytica* process of citric acid (CA) production [19,29]. In concert with the results obtained from the shake-flask experiments, overexpression of either hexokinase accelerated fructose utilization. It also led to higher CA secretion, most likely caused by debottlenecked glycolysis (Figure 5), as hexokinase is one of the major factors limiting this core pathway in *Y. lipolytica* [23,24]. The strains expressing transporters alongside SpHxk1 utilized glucose and fructose more rapidly than their YlHxk1 counterparts and generally secreted less CA. Low CA yields of the YlH1-Y3 and control JMY2900 strain could be explained by incomplete fructose utilization in the given timeframe (132 h). As stated, the SpHxk1-expressing strains secreted much less CA than could be expected, what indicated that the available carbon was redirected to other pathways. Nitrogen limitation in the applied conditions is known to direct the carbon flux either towards CA biosynthesis or lipid accumulation [30]. The analyzed mutants did not differ much in the generated biomass and lipid content, they had, however, produced significant amounts of polyols, of which mannitol and erythritol were predominant. The increased polyol secretion may have three possible explanations: (1) hexose transporters in *Y. lipolytica* have a dual function—hexose import and polyol export; (2) the limited capacity of glycolysis results in the redirection of carbon flux to other pathways, such as pentose phosphate pathway (Figure 5); (3) the high sugar influx results in rising osmotic pressure inside the cells, creating a need for a defense mechanism allowing for pressure equalization. While the first explanation is unlikely, as evidenced by the bioreactor experiment in which the YHT*1-4Δ* mutant secreted polyols (Figure S6), the second and third hypotheses are probable. Thus, carbon overflow through glycolysis can be compensated by directing the flux to pentose phosphate pathway, which results in increased erythritol biosynthesis. It would be also interesting to see, whether the elimination of more bottlenecks in glycolysis, for example by overexpression of *PFK1* (Figure 5), would push the carbon flux down the pathway to the TCA cycle instead of directing it towards pentose phosphates. At this point, it is worth mentioning that secreted CA can be re-incorporated into the metabolism when supplied carbon sources are depleted [19], which creates a need for careful bioprocess design. In turn, since it is known that *Y. lipolytica* secretes erythritol in response to the presence of glycerol [12], a similar mechanism might exist for sugar response. 

The rerouting of carbon excess toward polyol production led us to evaluate polyols production by our engineered strains. Specifically, we wondered whether the production capacities of the strains expressing SpHxk1 and YHT3 could be further improved by the optimization of culture conditions. For that purpose, we chose the SpH1-Y1-Y3-Y4 strain as it showed the fastest consumption of glucose and fructose accompanied by a significant secretion of polyols. The strain was cultivated in bioreactors in media optimized for polyol production [31], containing either a mixture of glucose and fructose or glycerol as a carbon source. In the conditions tested, *Y. lipolytica* SpH1-Y1-Y3-Y4 used glucose and fructose 24 h earlier than in the CA production media. At the same time, it secreted insignificant amounts of CA at an expense of increased polyol levels. For comparison, the strain was also cultivated on glycerol. The complete substrate exhaustion occurred 10 h later than in the hexose-based media and more CA was formed. The sum of produced polyols was lower; however, the obtained mixture was more homogenous as erythritol was predominant. The faster consumption of sugars in the optimized conditions could be explained by the higher availability of nitrogen in the medium, which translated into a higher growth rate [32]. In turn, glycerol could not be utilized as fast, as the strain did not contain any improvements in the glycerol assimilation pathway (Figure 5); however, its utilization rate was nearly identical to that reported previously for the wild-type *Y. lipolytica* strain (0.02 mol/L/h vs. 0.022 mol/L/h) [32]. The obtained quantities of particular polyols strictly depended on the used substrates. *Y. lipolytica* tends to overproduce erythritol in response to the osmotic pressure induced by glycerol [12], and the strain used in our work behaved in the same manner. On the other hand, when grown on glucose and fructose, mannitol was predominant, followed by significant amounts of erythritol and arabitol. In these conditions, increased mannitol biosynthesis could be expected as it is only one metabolic step from fructose (Figure 5), carried out by mannitol dehydrogenase [33,34], which also coincided with the period of high-rate fructose consumption.

We were curious to whether the polyol spectrum could be changed by further optimization of culture conditions. The optimization relied on the addition of NaCl to the previously used media to increase the osmotic pressure and induce erythritol secretion [31]. Glucose and fructose were used as quickly as before, but more biomass and only trace amounts of CA were made. As expected, the polyol spectrum changed in favor of erythritol, and mannitol was produced in very low quantities. Recently, Wang et al. (2020) [34] carried out metabolic engineering of *Y. lipolytica*, aiming to increase erythritol biosynthesis through the identification and subsequent suppression of the genes involved in mannitol and arabitol formation. They pointed out that the knock-out of the gene *MDH2* (*YALI0D18964g*) encoding mannitol dehydrogenase resulted in a complete loss of ability to produce mannitol, which accompanied a slight increase in erythritol production. We observed a similar phenomenon caused by the presence of salt in the medium. As evidenced by the gene expression data, NaCl (or increased osmotic pressure in general) caused a downregulation of *MDH2* (Figure S7).Although the titers of polyols, especially erythritol obtained in our study, cannot compete with those previously reported, reaching or even exceeding 200 g/L during growth in glycerol-based media [12,35], we find it interesting to see that it is possible to obtain decent amounts of these compounds using hexoses found in abundance in industrial wastes or lignocellulosic materials [17,36]. To the best of our knowledge, we show here for the first time, rapid polyol production by *Y. lipolytica* in fructose-containing media through the modifications of the upstream cellular processes, i.e., sugar import and activation. We reason that further engineering of metabolic pathways directly implicated in polyol biosynthesis, such as pentose phosphate pathway (Figure 5), could enhance the production capacities of the SpH1-Y1-Y3-Y4 strain, as it was previously reported in transketolase-(*TKL1*), erythrose reductase-(*ER*) or erythritol dehydrogenase-(*EYD1*) overexpressing *Y. lipolytica* strains [37,38,39,40]. As it is in the case of CA, secreted erythritol can also be catabolized by the *Y. lipolytica* cells when carbon sources are depleted, a process in which erythritol kinase encoded by the *EYK1* gene plays a major role [41]. Therefore, careful bioprocess characterizations, in which the exact time points of substrate exhaustion are provided, as presented in the present study, and/or the disruption of *EYK1* could lead to higher yields. From an industrial perspective, it would also be of great interest to analyze the performance of the SpH1-Y1-Y3-Y4 derivatives with overexpressed invertase [42] and modified downstream metabolic pathways in industrial setups, including the use of molasses.

## 4. Materials and Methods

### 4.1. Culture Media

The *Escherichia coli* strains were maintained in a LB medium, containing 5 g/L yeast extract (YE), 10 g/L tryptone, 10 g/L NaCl with 20 g/L agar in plates and 0.05 mg/L kanamycin for selection of transformants at 37 °C. *Y. lipolytica* strains were maintained in a YPG medium, consisting of 10 g/L yeast extract, 20 g/L peptone, 20 g/L glycerol, 20 g/L agar (for plates) and 0.2 g/L Hygromycin B (when applicable) at 28 °C. Minimal (YNB) medium for selection of *Y. lipolytica* transformants was prepared using a 1.9 g/L yeast nitrogen base (without amino acids and ammonium sulfate; Formedium Ltd., Hunstanton, UK), 10 g/L glycerol, 5 g/L NH_4_Cl, 50 mM phosphate buffer pH 6.8 with 20 g/L agar and 0.1 g/L uracil or 0.2 g/L leucine when needed. For long-term storage the strains were kept in 500 g/L glycerol at −80 °C. 

Minimal media with single sugar used for *S. cerevisiae* and *Y. lipolytica* substrate utilization kinetics and *Y. lipolytica* microcultivations contained 1.9 g/L YNB, 5 g/L NH_4_Cl, 50 mM phosphate buffer pH 6.8 and 10 g/L glucose, fructose or mannose. The medium with a mixture of glucose and fructose used in microcultivations and *S. cerevisiae* and *Y. lipolytica* substrate utilization kinetics consisted of 1.9 g/L YNB, 10 g/L NH_4_Cl, 50 mM phosphate buffer pH 6.8, 10 g/L glucose and 10 g/L fructose.

The medium for citric acid production contained 50 g glucose, 50 g fructose, 1.5 g NH_4_Cl, 0.7 g KH_2_PO_4_, 1.0 g MgSO_4_·7H_2_O, 0.3 g YE and 0.3 × 10^−3^ g thiamine in 1 L of tap water. The media optimized for the production of polyols and erythritol were adopted from [31]. The polyol production media consisted of a mixture of 50 g glucose and 50 g fructose or 100 g glycerol as the carbon source, 2.7 g (NH_4_)_2_SO_4_, 0.2 g KH_2_PO_4_, 1.0 g MgSO_4_·7H_2_O, 1.6 g YE in 1 L tap water with 3.0 g CaCO_3_ (for the shake-flask experiment). The erythritol media was composed of a mixture of 50 g glucose and 50 g fructose or 100 g glycerol, 4.6 g (NH_4_)_2_SO_4_, 0.22 g KH_2_PO_4_, 1.0 g MgSO_4_·7H_2_O, 1.0 g YE and 25.5 g NaCl in 1 L tap water with the addition of 3.0 g CaCO_3_ in the shake-flask experiment. Stocks of sugars and glycerol were of concentration 500 g/L and cold-sterilized through 0.22 µm membranes. The remaining media components were autoclaved at 121 °C for 20 min. 

### 4.2. Strain Construction

The *S. cerevisiae* strains expressing hexose transporters from *Y. lipolytica* were constructed in our previous study [20].

**Figure 6:**
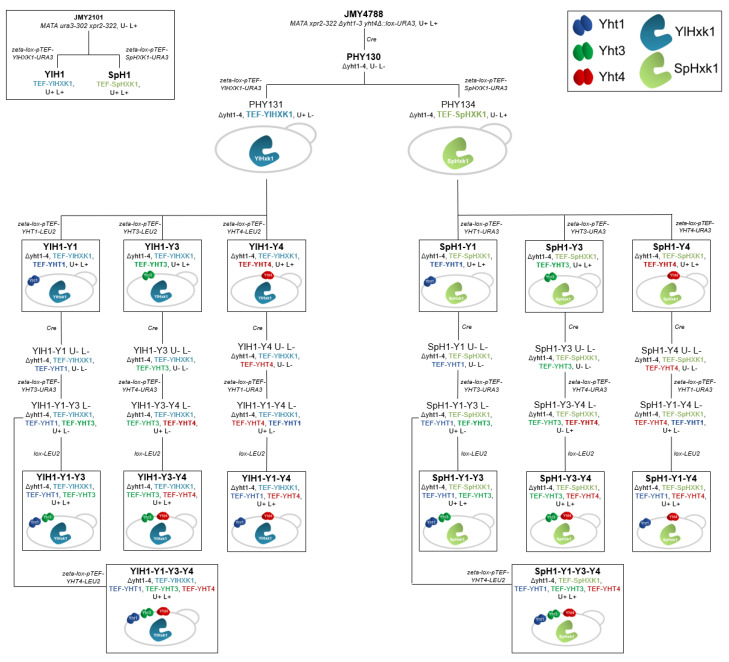
Construction of *Y. lipolytica* strains expressing hexokinases and hexose transporters. A detailed list of the *Y. lipolytica* strains used in this study is available in Table S4.

The transporter and hexokinase genes from *Y. lipolytica* were amplified from a wild-type W29 strain genome using Phusion High-Fidelity DNA polymerase (Thermo Scientific, Waltham, MA, USA) and primers listed in Table S1 with overhangs containing *Bam*HI (or *Bgl*II) and *Avr*II recognition sites. Amplified DNA fragments were then gel-purified using the Gel-Out Concentrator kit (A&A Biotechnology, Gdynia, Poland) and digested with appropriate restriction enzymes. Prepared fragments were then cloned into *Bam*HI/*Avr*II digested and dephosphorylated JMP62-URA3ex-pTEF or JMP62-LEU2ex-pTEF vectors containing *zeta* sequences for random chromosomal integration [43]. The vectors were then used for transformation of competent *E. coli* DH5α strain. The colonies were verified using the PCR-colony technique and Sanger sequencing (Genomed S.A., Warsaw, Poland). Correct vectors were then digested with *Not*I to release expression cassettes and were used for *Y. lipolytica* transformations. All the *Y. lipolytica* transformations were performed using the lithium acetate method [44]. *SpHXK1* from *S. pombe* was obtained as a synthetic gene codon-optimized for expression in *Y. lipolytica* (GenScript, Leiden, the Netherlands) containing *Bam*HI/*Avr*II overhangs, cloned into JMP62-URA3ex-pTEF or JMP62-LEU2ex-pTEF vectors and used for *Y. lipolytica* transformations (Figure 6, Table S4 [45,46]). The obtained transformants were analyzed using PCR on genomic DNA extracted using the Genomic Mini AX Yeast kit (A&A Biotechnology) with primers listed in Table S1. The nucleotide sequence of optimized *SpHXK1* is given in Supplementary File S1. The deduced amino acid sequence is identical to the NCBI Reference Sequence NP_592948.1. 

The *Y. lipolytica* strains were constructed in an YHT*1-4Δ* genetic background in which native hexokinase and glucokinase were intact (*Y. lipolytica* JMY4788; [20]). The markers in this strain were rescued using CreLox recombinase [43]. Next, two hexokinases, native *YlHXK1* (*YALI0B22308g*) and heterologous *SpHXK1* were overexpressed, creating a starting point for two branches of subsequent transformants (Figure 6). These two strains were further engineered by the overexpression of single (*YHT1 (YALI0C06424g)*, *YHT3 (YALI0F19184g)*, *YHT4 (YALI0E23287g)*), combinations of two (*YHT1*-*YHT3*, *YHT1*-*YHT4*, *YHT3*-*YHT4*) and three (*YHT1*-*YHT3*-*YHT4*) transporter genes using the *Not*I-digested JMP62-URA3ex-pTEF- or JMP62-LEU2ex-pTEF-based vectors. The obtained transformants were analyzed using PCR on genomic DNA. In each branch there was one step required for marker rescue and one for prototrophy restoration using the *LEU2ex* fragment obtained from JMP62-LEU2ex-pTEF plasmid via I-*Sce*I digestion. Due to the randomness of the genome integration site and possible phenotypic variations resulting from the nature of the employed overexpression method, three strains of each genotype were used for the initial experiments. 

### 4.3. Microcultivations

For growth profiling of *Y. lipolytica* transformants growing on glucose, fructose and a mixture of both in minimal YNB media a Synergy H1 microplate reader (BioTek, Winooski, VT, USA) was used. Prior to the experiment the cells were grown in 5 mL YPG medium overnight at 28 °C and 180 rpm, washed thrice with sterile distilled water and standardized to OD10. Cultivations were carried out in 96-well microtiter plates (NEST, Wuxi, China) with a working volume of 200 µL. Initial OD was set to 0.5. Throughout the process, the temperature was maintained at 28 °C and 600 rpm linear shaking was applied. The growth was monitored by measuring optical density in 10 min intervals. The experiments were conducted in three biological replicates.

### 4.4. Substrate Utilization Kinetics

The substrate utilization kinetics were carried out in 250 mL Erlenmeyer flasks with 50 mL YNB medium with a mixture of glucose and fructose. Prior to inoculation, the cells were prepared as described in Section 4.3. Prepared media were inoculated to an OD 0.5 and incubated at 28 °C on a rotary shaker with 180 rpm shaking speed. The samples were taken in 3 h intervals for growth and substrate utilization analysis. The experiments were conducted in three biological replicates and standard deviations were calculated. 

### 4.5. Bioreactor Cultivations

The bioreactor cultivations were carried out in 5 L stirred-tank BIOSTAT B plus reactors (Sartorius AG, Frankfurt, Germany). The bioreactors with appropriate media were sterilized at 121 °C for 20 min. Carbon sources were cold-sterilized and added directly to the vessels after sterilization in autoclaves. Prior to inoculations, the cells were grown in 50 mL YPG medium in 250 mL Erlenmeyer flasks at 28 °C, 180 rpm overnight and subsequently washed thrice with sterile distilled water. The initial OD was set to 0.5 and the working volume was 2 L. The cultivations were conducted at 28 °C with a stirring rate of 800 rpm and an aeration rate of 0.8 vvm (vessel volume per minute). pH was automatically maintained at 6.8 (for citric acid production) or 3 (for polyols) using 40% (*w*/*v*) NaOH solution. All the bioreactor experiments were conducted in three biological replicates and standard deviations were calculated.

### 4.6. Analytical Methods

The optical density measurements in the kinetic experiments were carried out using the SmartSpec Plus spectrophotometer (Biorad, Hercules, CA, USA). The dry biomass during bioreactor cultures was determined gravimetrically after cells being filtered through 0.22 μm membranes and dried at 105 °C using laboratory scale with moisture analyzer (RADWAG, Radom, Poland).

The concentrations of glucose, fructose, mannose, glycerol, citric acid and polyols were determined using the Dionex UltiMate 3000 HPLC instrument (Dionex-Thermo Fisher, Sunnyvale, CA, USA) equipped with a Carbohydrate H+ column (Thermo Scientific) coupled to UV (λ = 210 nm) and RI (Shodex, Ogimachi, Japan) detectors. The column was eluted with 25 mM trifluoroacetic acid (TFA) at 65 °C and a flow rate of 0.6 mL/min. 

A two-step direct transesterification method [47] was used for lipid quantification. Briefly, 1 mg of biomass was collected, centrifuged for 2 min at 8000 rpm and the supernatant was discarded. Next, 100 µL of C17:0 internal standard (Sigma-Aldrich, St. Louis, MO, USA) was added to the samples. After the addition of 500 µL 0.5 M sodium methoxide, the samples were vortexed for 60 min at room temperature. Following incubation, 40 µL anhydrous H_2_SO_4_ and 1 mL hexane were added and left overnight at room temperature. After centrifugation at 8000 rpm for 1 min, the upper hexane layer containing fatty acid methyl esters (FAMEs) was collected and analyzed by GC-MS instrument (Shimadzu, Kyoto, Japan) equipped with a Zebron ZB-FAME capillary column (30 m × 0.25 mm × 0.20 µm). The samples (1 µL at 250 °C) were injected in splitless mode using helium (1 mL/min). Identification of fatty acids was carried out by a comparison of retention times with reference compounds (Supelco 37 Component FAME Mix, Sigma-Aldrich). 

### 4.7. Gene Expression Analysis

RNA from the collected samples was immediately extracted using the Total RNA Mini kit (A&A Biotechnology, Gdynia, Poland) according to the supplied protocol and its concentration and quality was verified using the Biochrom WPA Biowave DNA spectrophotometer (Biochrom Ltd., Cambridge, UK) Extracted RNA samples were treated with DNase (A&A Biotechnology, Gdynia, Poland) and reverse transcribed to cDNA using Maxima First Strand cDNA Synthesis Kit for RT-qPCR (Thermo Scientific). The obtained cDNA samples were then used for qPCR reaction using the Maxima SYBR Green qPCR Master Mix (Thermo Scientific) and primers listed in Table S1 in the PCRmax Eco 48 thermal cycler (Illumina, San Diego, CA, USA). The expression of analyzed genes was standardized to the expression of the actin (*YlACT1*, *YALI0D08272g*) gene. The gene expression levels were examined in three biological replicates.

### 4.8. Calculations 

Substrate utilization rates (r), expressed as gram of substrate per liter per hour, were calculated for linear utilization curves using following equation:

(1) $$r=\frac{S_{f}-S_{i}}{t_{f}-t_{i}}$$

where S_i_ and S_f_ are substrate concentrations at the beginning and end of the linear substrate utilization, respectively, [g/L] and t_f_ and t_i_ represent time of culture at the beginning and end of the linear substrate utilization, respectively [h].

Maximum growth rates (gram of biomass per liter per hour) were calculated as follows:

(2) $$r_{\mathrm{xmax}}=\frac{X_{f}-X_{i}}{t_{f}-t_{i}}$$

where X_i_ and X_f_ are biomass concentrations at the beginning and end of the logarithmic growth phase, respectively, [g/L] and t_f_ and t_i_ represent time of culture at the beginning and end of the logarithmic growth phase, respectively [h].

The biomass yield from a gram of substrate (Y_X/S_) was determined using:

(3) $$Y=\frac{X}{S}$$

The mass yield coefficient of product formation (Y_P/S_), expressed as grams of product per gram of consumed substrate, was calculated from the following:

(4) $$Y=\frac{P}{S}$$

The specific productivity (q, grams of product per gram of biomass per hour) was calculated with:

(5) $$q=\;\frac{P}{X*t}$$

The volumetric productivity (Q, grams of product per liter per hour) was calculated using:

(6) $$Q=\;\frac{P}{V*t}$$

where P is the total amount of product in the culture medium at the end of cultivation [g], S is the total amount of the consumed substrate [g], V is the initial volume of culture [L], X is the biomass concentration [g] and t is the time of cultivation [h].

## 5. Conclusions

In the present study, we intended to point out molecular actors responsible for preferential glucose over fructose uptake in the oleaginous yeast *Y. lipolytica*. Specifically, we chose proteins responsible for sugar transport and activation, and by combining a variety of experimental methods, including heterologous expression, time-course experiments and bioreactor studies, we showed that the interplay between hexose transporters and hexokinase is implicated in the preferred glucose consumption. However, in order to recognize the true nature of these interactions on glucose preference, future studies with *Y. lipolytica* mutants devoid of glucokinase are necessary. Additionally, we indicated that the input flux of carbon has a large impact on downstream metabolic pathways and that this flux can be directed to the molecules of industrial significance.

## Figures and Tables

**Table 1:** Parameters of bioreactor cultures for citric acid production using *Y. lipolytica* transformants in defined medium with a mixture of glucose and fructose. The cultivations were carried out until complete depletion of the substrates or until 132 h (strains JMY2900, YlH1-Y3 and YlH1-Y3-Y4) and were performed in three biological replicates.

Parameter	Unit	JMY2900	YlH1	SpH1	YlH1-Y3	YlH1-Y1-Y3	YlH1-Y3-Y4	YlH1-Y1-Y3-Y4	SpH1-Y3	SpH1-Y1-Y3	SpH1-Y3-Y4	SpH1-Y1-Y3-Y4
r_xmax_	g/L/h	0.13	0.14	0.08	0.10	0.07	0.08	0.24	0.17	0.19	0.18	0.22
r_glc_		0.73	1.04	1.03	1.09	1.26	1.86	1.35	1.16	1.53	2.51	1.35
r_fru_		0.43	0.84	0.77	0.41	0.91	0.89	1.77	1.34	1.43	1.70	1.67
X	g/L	17.4 ± 1.2	14.5 ± 1.0	10.5 ± 0.9	13.5 ± 0.3	8.0 ± 1.0	10.5 ± 1.4	17.2 ± 2.4	11.9 ± 0.8	13.0 ± 1.3	12.6 ± 1.2	14.0 ± 1.8
CA		29.39 ± 2.33	67.90 ± 3.51	81.09 ± 4.55	26.45 ± 1.24	65.50 ± 4.33	70.19 ± 2.22	36.59 ± 4.22	42.84 ± 1.83	41.87 ± 1.97	35.78 ± 1.76	33.19 ± 2.40
Lipids		5.74 ± 1.35	4.42 ± 1.00	4.02 ± 0.98	3.23 ± 1.33	3.46 ± 1.03	3.61 ± 1.22	3.97 ± 1.27	2.83 ± 0.66	3.51 ± 0.83	3.51 ± 0.97	3.08 ± 1.56
Man		ND	1.34 ± 0.27	ND	ND	ND	1.93 ± 0.06	8.47 ± 1.03	8.47 ± 0.98	13.26 ± 0.56	13.56 ± 0.96	13.42 ± 1.88
Ara		ND	0.90 ± 0.03	ND	ND	ND	0.78 ± 0.13	4.98 ± 0.52	4.10 ± 0.08	3.02 ± 0.22	4.08 ± 1.03	3.20 ± 0.06
Ery		ND	ND	ND	ND	ND	ND	7.87 ± 1.12	11.94 ± 1.16	9.03 ± 1.09	13.84 ± 1.69	9.34 ± 0.97
Sum of polyols		ND	2.24	ND	ND	ND	2.71	21.32	24.51	25.31	31.48	25.96
Y_CA/S_	g/g	0.427	0.728	0.804	0.420	0.667	0.751	0.398	0.447	0.438	0.361	0.353
Q_CA_	g/L/h	0.111	0.314	0.307	0.100	0.287	0.266	0.254	0.298	0.303	0.248	0.263
q_CA_	g/g/h	0.013	0.043	0.060	0.015	0.072	0.051	0.030	0.050	0.047	0.039	0.038

Abbreviations: Ara—arabitol; CA—citric acid; Ery—erythritol; Man—mannitol; ND—not detected; Q_CA—_CA volumetric productivity; q_CA_—CA specific productivity; r_fru_—maximum fructose utilization rate; r_glc_—maximum glucose utilization rate; r_xmax_—maximum growth rate; S—sum of glucose and fructose concentrations at the beginning of cultivation; X—biomass; Y—yield.

**Table 2:** Parameters of bioreactor cultures for polyol biosynthesis using *Y. lipolytica* SpH1-Y1-Y3-Y4 in media containing a mixture of glucose and fructose or glycerol. The cultivations were carried out until complete depletion of the substrate(s) and were performed in three biological replicates.

Parameter	Unit	Polyol Media		Erythritol Media	
		Glucose Fructose	Glycerol	Glucose Fructose	Glycerol
r_xmax_	g/L/h	0.693	0.857	0.767	0.400
r_glc_		2.048	-	2.738	-
r_fru_		2.698	-	3.229	-
r_gly_		-	2.432	-	2.013
Man	g/L	18.03 ± 1.37	5.04 ± 0.90	2.09 ± 0.66	0.11 ± 0.01
Ara		8.48 ± 0.68	4.29 ± 1.02	14.66 ± 1.06	4.07 ± 0.32
Ery		14.07 ± 1.29	24.28 ± 0.99	23.12 ± 1.43	28.31 ± 1.33
Y_Man/S_	g/g	0.199	0.049	0.021	0.001
Y_Ara/S_		0.094	0.041	0.145	0.037
Y_Ery/S_		0.155	0.235	0.229	0.257
Y_Man/X_		1.050	0.319	0.101	0.007
Y_Ara/X_		0.493	0.271	0.708	0.238
Y_Ery/X_		0.819	1.534	1.117	1.655
q_Man_	g/g/h	0.022	0.012	0.002	0.000
q_Ara_		0.010	0.006	0.015	0.003
q_Ery_		0.017	0.018	0.023	0.020
Q_Man_	g/L/h	0.188	0.043	0.022	0.000
Q_Ara_		0.088	0.037	0.153	0.024
Q_Ery_		0.147	0.209	0.241	0.169

Abbreviations: Ara—arabitol; Ery—erythritol; Man—mannitol; Q—volumetric productivity; q—CA specific productivity; rfru—maximum fructose utilization rate; r_glc_—maximum glucose utilization rate; r_gly_—maximum glycerol utilization rate; r_xmax_—maximum growth rate; S—sum of substrate concentrations at the beginning of cultivation; X—biomass; Y—yield.

## Supplementary Materials

The following are available online at https://www.mdpi.com/article/10.3390/ijms22179282/s1.
