# Supplementary material for: The Role of Hexokinase and Hexose Transporters in Preferential Use of Glucose over Fructose and Downstream Metabolic Pathways in the Yeast Yarrowia lipolytica
Source: Int J Mol Sci. 2021 Aug 27;22(17):9282. doi: 10.3390/ijms22179282 (PMC8431455; doi:10.3390/ijms22179282)
Supplement: Supplementary file 1 [file ijms-22-09282-s001.zip › ijms-1333919-Table_S1.pdf]

Table S1. Primers used in this study

Primers for gene overexpression	
YHT1-F-BglII	GTGAAGATCTATGGGACTCGCTAACATCATCAAC
YHT1-R-AvrII	GAGACCTAGGCTAGACAGACTCAATGTAGACGTGC
YHT3-F-BamHI	GTGAGGATCCATGTCCACTAGTGCTATGACCG
YHT3-R-AvrII	GAGACCTAGGCTAAGAGGACTCGGAGAAGTCG
YHT4-F-BamHI	GAGTGGATCCATGTACAAGGTCCACAACCCC
YHT4-R-AvrII	GAGACCTAGGTTAAACAGTCTCGGTGTACTGAGG
JMP62-pTEF-START	GGGTATAAAAGACCACCGTCC
JMP62-61STOP	GTAGATAGTTGAGGTAGAAGTTG
qPCR primers	
qACT1_F	CAAGCGAGGTATCCTGACCC
qACT1_R	CGGTTGGACTTGGGGTTGAT
qMDH1_F	GGTGCCTATTATTGCGCCAAG
qMDH1_R	ATCGGGACAGATGGAGGACA
qMDH2_F	GGCCACATTGTCAACATCCC
qMDH2_R	TCTCCTTGGGGACAAAGTCG
qTKL1_F	ATGGGAGAAGGCTCTGCCTA
qTKL1_R	GCTGGAAATCAACAGCGTCC
qER_F	CCGTTGTTTTCAAGACCGGC
qER_R	TCTCAATTCGCTCCTCGGTG
