# Supplementary material for: The Role of Hexokinase and Hexose Transporters in Preferential Use of Glucose over Fructose and Downstream Metabolic Pathways in the Yeast Yarrowia lipolytica
Source: Int J Mol Sci. 2021 Aug 27;22(17):9282. doi: 10.3390/ijms22179282 (PMC8431455; doi:10.3390/ijms22179282)
Supplement: Supplementary file 1 [file ijms-22-09282-s001.zip › ijms-1333919-Table_S2.pdf]

Table S2. Parameters of bioreactor cultures for citric acid production using *Y. lipolytica* transformants in defined medium with a mixture of glucose and fructose. The cultivations were carried out until complete depletion of the substrates or until 132 hours (strains JMY2900, YlH1-Y3 and YlH1-Y3-Y4) and were performed in three biological replicates.

Parameter	Unit	JMY2900	YlH1	SpH1	YlH1-Y1	SpH1-Y1	YlH1-Y3	SpH1-Y3	YlH1-Y4	SpH1-Y4	YlH1-Y1-Y3	SpH1-Y1-Y3	YlH1-Y1-Y4	SpH1-Y1-Y4	YlH1-Y3-Y4	SpH1-Y3-Y4	YlH1-Y1-Y3-Y4	SpH1-Y1-Y3-Y4
X		17.4 ± 1.2	14.5 ± 1.0	10.5 ± 0.9	14.5 ± 1.5	14.6 ± 2.0	13.5 ± 0.3	11.9 ± 0.8	14.8 ± 1.1	13.7 ± 0.7	8.0 ± 1.0	13.0 ± 1.3	13.8 ± 0.9	14.6 ± 0.6	10.5 ± 1.4	12.6 ± 1.2	17.2 ± 2.4	14.0 ± 1.8
CA		29.39 ± 2.33	67.90 ± 3.51	81.09 ± 4.55	52.02 ± 3.12	48.50 ± 2.21	26.45 ± 1.24	42.84 ± 1.83	40.70 ± 4.33	38.66 ± 2.34	65.50 ± 4.33	41.87 ± 1.97	46.31 ± 1.32	35.03 ± 1.99	70.19 ± 2.22	35.78 ± 1.76	36.59 ± 4.22	33.19 ± 2.40
Lipids		5.74 ± 1.35	4.42 ± 1.00	4.02 ± 0.98	3.64 ± 0.35	3.62 ± 0.88	3.23 ± 1.33	2.83 ± 0.66	3.55 ± 0.99	3.89 ± 0.76	3.46 ± 1.03	3.51 ± 0.83	3.56 ± 0.94	3.09 ± 1.11	3.61 ± 1.22	3.51 ± 0.97	3.97 ± 1.27	3.08 ± 1.56
Man	g/L	ND	1.34 ± 0.27	ND	6.73 ± 0.41	8.72 ± 0.78	ND	8.47 ± 0.98	9.52 ± 1.33	20.80 ± 2.63	ND	13.26 ± 0.56	12.17 ± 1.45	23.37 ± 1.87	1.93 ± 0.06	13.56 ± 0.96	8.47 ± 1.03	13.42 ± 1.88
Ara		ND	0.90 ± 0.03	ND	3.54 ± 0.66	2.79 ± 0.12	ND	4.10 ± 0.08	4.33 ± 0.94	4.27 ± 1.15	ND	3.02 ± 0.22	2.17 ± 0.44	4.12 ± 0.99	0.78 ± 0.13	4.08 ± 1.03	4.98 ± 0.52	3.20 ± 0.06
Ery		ND	ND	ND	4.50 ± 0.09	2.29 ± 0.21	ND	11.94 ± 1.16	11.01 ± 1.03	0.68 ± 0.21	ND	9.03 ± 1.09	3.93 ± 0.20	7.55 ± 1.05	ND	13.84 ± 1.69	7.87 ± 1.12	9.34 ± 0.97
Sum of polyols		ND	2.24	ND	14.77	13.80	ND	24.51	24.86	25.75	ND	25.31	18.28	35.04	2.71	31.48	21.32	25.96
$Y_{CA/S}$		0.427	0.728	0.804	0.547	0.513	0.420	0.447	0.408	0.383	0.667	0.438	0.517	0.347	0.751	0.361	0.398	0.353
$Y_{lipids/S}$		0.083	0.047	0.040	0.038	0.038	0.051	0.030	0.035	0.038	0.035	0.037	0.035	0.031	0.035	0.035	0.043	0.033
$Y_{Man/S}$		ND	0.014	ND	0.071	0.092	ND	0.088	0.095	0.206	ND	0.139	0.120	0.232	0.021	0.137	0.103	0.143
$Y_{Ara/S}$		ND	0.010	ND	0.037	0.029	ND	0.043	0.043	0.042	ND	0.032	0.021	0.041	0.008	0.041	0.054	0.034
$Y_{Ery/S}$		ND	ND	ND	0.024	0.024	ND	0.124	0.110	0.007	ND	0.095	0.039	0.075	ND	0.140	0.086	0.099
$Y_{polyols/S}$	g/g	ND	0.024	ND	0.155	0146	ND	0.256	0.250	0.255	ND	0.265	0.181	0.347	0.029	0.318	0.232	0.276
$Y_{X/S}$		0.253	0.156	0.104	0.152	0.154	0.214	0.124	0.148	0.136	0.081	0.136	0.136	0.145	0.112	0.127	0.187	0.149
$Y_{CA/X}$		1.689	4.583	7.723	3.588	3.322	1.959	3.600	2.750	2.822	8.188	3.221	3.356	2.399	6.685	2.840	2.127	2.371
$Y_{lipids/X}$		0.330	0.305	0.383	0.251	0.221	0.239	0.238	0.240	0.284	0.433	0.270	0.258	0.212	0.344	0.279	0.231	0.220
$Y_{Man/X}$		ND	0.092	ND	0.464	0.597	ND	0.712	0.643	1.518	ND	1.020	0.881	1.601	0.184	1.076	0.492	0.959
$Y_{Ara/X}$		ND	0.062	ND	0.244	0.191	ND	0.344	0.293	0.312	ND	0.232	0.157	0.282	0.074	0.324	0.290	0.229

$Y_{Ery/X}$		ND	ND	ND	0.310	0.157	ND	1.003	0.744	0.050	ND	0.695	0.285	0.517	ND	1.100	0.458	0.667
$Y_{polyols/X}$		ND	0.154	ND	1.019	0.945	ND	2.060	1.680	1.888	ND	1.947	1.325	2.400	0.258	2.498	1.240	1.854
q_{CA}	g/g/h	0.013	0.043	0.060	0.043	0.040	0.015	0.050	0.038	0.039	0.072	0.047	0.047	0.033	0.051	0.039	0.030	0.038
q_{lipids}		0.002	0.003	0.003	0.003	0.003	0.002	0.003	0.003	0.004	0.004	0.004	0.004	0.003	0.003	0.004	0.003	0.003
q_{Man}		ND	0.001	ND	0.006	0.007	ND	0.010	0.009	0.021	ND	0.014	0.012	0.022	0.001	0.015	0.007	0.015
q_{Ara}		ND	0.001	ND	0.003	0.002	ND	0.005	0.004	0.004	ND	0.003	0.002	0.004	0.001	0.004	0.004	0.004
q_{Ery}		ND	ND	ND	0.004	0.002	ND	0.014	0.010	0.001	ND	0.010	0.004	0.007	ND	0.015	0.006	0.011
$q_{polyols}$		ND	0.001	ND	0.012	0.011	ND	0.029	0.023	0.026	ND	0.028	0.018	0.033	0.002	0.035	0.017	0.029
Q_{CA}	g/L/h	0.111	0.314	0.307	0.310	0.289	0.100	0.298	0.283	0.268	0.287	0.303	0.322	0.243	0.266	0.248	0.254	0.263
Q_{lipids}		0.022	0.020	0.015	0.022	0.022	0.012	0.020	0.025	0.027	0.015	0.025	0.025	0.021	0.025	0.024	0.028	0.024
Q_{Man}		ND	0.006	ND	0.040	0.052	ND	0.059	0.066	0.145	ND	0.096	0.085	0.162	0.007	0.094	0.059	0.107
Q_{Ara}		ND	0.004	ND	0.021	0.017	ND	0.028	0.030	0.030	ND	0.022	0.015	0.029	0.003	0.028	0.035	0.025
Q_{Ery}		ND	ND	ND	0.027	0.014	ND	0.083	0.076	0.076	ND	0.065	0.027	0.052	ND	0.096	0.055	0.074
$Q_{polyols}$		ND	0.010	ND	0.088	0.082	ND	0.170	0.173	0.179	ND	0.183	0.127	0.243	0.010	0.219	0.148	0.206

Abbreviations: Ara - arabitol; CA - citric acid; Ery - erythritol; Man - mannitol; ND - not detected; Q_{CA} - CA volumetric productivity; q_{CA} - CA specific productivity; r_{fru} - maximum fructose utilization rate; r_{glc} - maxium glucose utilization rate; r_{xmax} - maximum growth rate; S - sum of glucose and fructose concentrations at the beginning of cultivation; X- biomass; Y - yield.
