# Supplementary material for: The Role of Hexokinase and Hexose Transporters in Preferential Use of Glucose over Fructose and Downstream Metabolic Pathways in the Yeast Yarrowia lipolytica
Source: Int J Mol Sci. 2021 Aug 27;22(17):9282. doi: 10.3390/ijms22179282 (PMC8431455; doi:10.3390/ijms22179282)
Supplement: Supplementary file 1 [file ijms-22-09282-s001.zip › ijms-1333919-Table_S3.pdf]

Table S3. Parameters of bioreactor cultures for polyol biosynthesis using *Y. lipolytica* SpH1-Y1-Y3-Y4 in media containing a mixture of glucose and fructose or glycerol. The cultivations were carried out until complete depletion of the substrate(s) and performed in three biological replicates.

Parameter	Unit	Polyol media		Erythritol media	
		Glucose + fructose	Glycerol	Glucose Fructose	Glycerol
X	g/L	17.2 ± 1.2	15.8 ± 1.6	20.7 ± 2.0	17.1 ± 1.5
r_{glc}	g/L/h	2.048	-	2.738	-
r_{fru}		2.698	-	3.229	-
r_{gly}		-	2.432	-	2.013
Man	g/L	18.03 ± 1.37	5.04 ± 0.90	2.09 ± 0.66	0.11 ± 0.01
Ara		8.48 ± 0.68	4.29 ± 1.02	14.66 ± 1.06	4.07 ± 0.32
Ery		14.07 ± 1.29	24.28 ± 0.99	23.12 ± 1.43	28.31 ± 1.33
Sum of polyols		40.58	33.61	39.87	32.49
CA		0.47 ± 0.06	2.09 ± 0.35	0.29 ± 0.04	5.51 ± 0.74
$Y_{Polyols/S}$	g/g	0.376	0.325	0.394	0.295
$Y_{Man/S}$		0.199	0.049	0.021	0.001
$Y_{Ara/S}$		0.094	0.041	0.145	0.037
$Y_{Ery/S}$		0.155	0.235	0.229	0.257
$Y_{CA/S}$		0.014	0.020	0.003	0.050
$Y_{X/S}$		0.190	0.153	0.205	0.155
$Y_{Polyols/X}$		2.362	2.124	1.926	1.900
$Y_{Man/X}$		1.050	0.319	0.101	0.007
$Y_{Ara/X}$		0.493	0.271	0.708	0.238
$Y_{Ery/X}$		0.819	1.534	1.117	1.655
$Y_{CA/X}$		0.027	0.132	0.014	0.322
q_{Man}	g/g/h	0.022	0.005	0.002	0.000
q_{Ara}		0.010	0.005	0.015	0.003
q_{Ery}		0.017	0.026	0.023	0.020
$q_{Polyols}$		0.049	0.036	0.040	0.023
q_{CA}		0.001	0.002	0.000	0.004
Q_{Man}	g/L/h	0.188	0.043	0.022	0.000
Q_{Ara}		0.088	0.037	0.153	0.024
Q_{Ery}		0.147	0.209	0.241	0.169
$Q_{Polyols}$		0.423	0.290	0.415	0.193
Q_{CA}		0.005	0.018	0.003	0.033

Abbreviations: Ara - arabitol; CA - citric acid; Ery - erythritol; Man - mannitol; Q - volumetric productivity; q - CA specific productivity; r_{fru} - maximum fructose utilization rate; r_{glc} - maxium glucose utilization rate; r_{gly} - maximum glycerol utilization rate; r_{xmax} - maximum growth rate; S - sum of substrate concentrations at the beginning of cultivation; X- biomass; Y - yield.
