# Supplementary material for: The Role of Hexokinase and Hexose Transporters in Preferential Use of Glucose over Fructose and Downstream Metabolic Pathways in the Yeast Yarrowia lipolytica
Source: Int J Mol Sci. 2021 Aug 27;22(17):9282. doi: 10.3390/ijms22179282 (PMC8431455; doi:10.3390/ijms22179282)
Supplement: Supplementary file 1 [file ijms-22-09282-s001.zip › ijms-1333919-Table_S4.pdf]

Table S4. *Y. lipolytica* strains used in this study.

Strain	Genotype or other relevant characteristics	Reference
JMY2101	*MATa ura3-302 leu2-270 xpr2-322,* Ura^{-} Leu^{+}	[45]
JMY2900	*MATa ura3-302 leu2-270, xpr2-322,* Ura^{+} Leu^{+}	[46]
JMY4788	*MATA xpr2-322 Δyht1-3 yht4Δ::lox-URA3,* Ura^{+} Leu^{+}	[20]
YlH1	JMY2101, *zeta-lox-pTEF-YlHXK1-URA3*, Ura^{+} Leu^{+}	This work
SpH1	JMY2101, *zeta-lox-pTEF-SpHXK1-URA3*, Ura^{+} Leu^{+}	
PHY130	JMY4788, Ura^{-} Leu^{-}	This work
PHY131	PHY130, *zeta-lox-pTEF-YlHXK1-URA3*, Ura^{+} Leu^{-}	
PHY134	PHY130, *zeta-lox-pTEF-SpHXK1-LEU2*, Ura^{-} Leu^{+}	
YlH1-Y1	PHY131, *zeta-lox-pTEF-YHT1-LEU2*, Ura^{+} Leu^{+}	
YlH1-Y3	PHY131, *zeta-lox-pTEF-YHT3-LEU2*, Ura^{+} Leu^{+}	
YlH1-Y4	PHY131, *zeta-lox-pTEF-YHT4-LEU2*, Ura^{+} Leu^{+}	
YlH1-Y1 U- L-	YlH1-Y1, Ura^{-} Leu^{-}	
YlH1-Y3 U- L-	YlH1-Y3, Ura^{-} Leu^{-}	
YlH1-Y4 U- L-	YlH1-Y4, Ura^{-} Leu^{-}	
YlH1-Y1-Y3 L-	YlH1-Y1 U- L-, *zeta-lox-pTEF-YHT3-URA3*, Ura^{+} Leu^{-}	
YlH1-Y3-Y4 L-	YlH1-Y3 U- L-, *zeta-lox-pTEF-YHT4-URA3*, Ura^{+} Leu^{-}	
YlH1-Y1-Y4 L-	YlH1-Y4 U- L-, *zeta-lox-pTEF-YHT1-URA3*, Ura^{+} Leu^{-}	
YlH1-Y1-Y3	YlH1-Y1-Y3 L-, *lox-LEU2*, Ura^{+} Leu^{+}	
YlH1-Y3-Y4	YlH1-Y3-Y4 L-, *lox-LEU2*, Ura^{+} Leu^{+}	
YlH1-Y1-Y4	YlH1-Y1-Y4 L-, *lox-LEU2*, Ura^{+} Leu^{+}	
YlH1-Y1-Y3-Y4	YlH1-Y1-Y3 L-, *zeta-lox-pTEF-YHT4-LEU2*, Ura^{+} Leu^{+}	
SpH1-Y1	PHY134, *zeta-lox-pTEF-YHT1-URA3*, Ura^{+} Leu^{+}	
SpH1-Y3	PHY134, *zeta-lox-pTEF-YHT3-URA3*, Ura^{+} Leu^{+}	
SpH1-Y4	PHY134, *zeta-lox-pTEF-YHT4-URA3*, Ura^{+} Leu^{+}	
SpH1-Y1 U- L-	SpH1-Y1, Ura^{-} Leu^{-}	
SpH1-Y3 U- L-	SpH1-Y3, Ura^{-} Leu^{-}	
SpH1-Y4 U- L-	SpH1-Y4, Ura^{-} Leu^{-}	
SpH1-Y1-Y3 L-	SpH1-Y1 U- L-, *zeta-lox-pTEF-YHT3-URA3*, Ura^{+} Leu^{-}	
SpH1-Y3-Y4 L-	SpH1-Y3 U- L-, *zeta-lox-pTEF-YHT4-URA3*, Ura^{+} Leu^{-}	
SpH1-Y1-Y4 L-	SpH1-Y4 U- L-, *zeta-lox-pTEF-YHT1-URA3*, Ura^{+} Leu^{-}	
SpH1-Y1-Y3	SpH1-Y1-Y3 L-, *lox-LEU2*, Ura^{+} Leu^{+}	
SpH1-Y3-Y4	SpH1-Y3-Y4 U-, *lox-LEU2*, Ura^{+} Leu^{+}	
SpH1-Y1-Y4	SpH1-Y1-Y4 L-, *lox-LEU2*, Ura^{+} Leu^{+}	
SpH1-Y1-Y3-Y4	SpH1-Y1-Y3 L-, *zeta-lox-pTEF-YHT4-LEU2*, Ura^{+} Leu^{+}	
