# Supplementary figures and images for: The Role of Hexokinase and Hexose Transporters in Preferential Use of Glucose over Fructose and Downstream Metabolic Pathways in the Yeast Yarrowia lipolytica
Source: Int J Mol Sci. 2021 Aug 27;22(17):9282. doi: 10.3390/ijms22179282 (PMC8431455; doi:10.3390/ijms22179282)

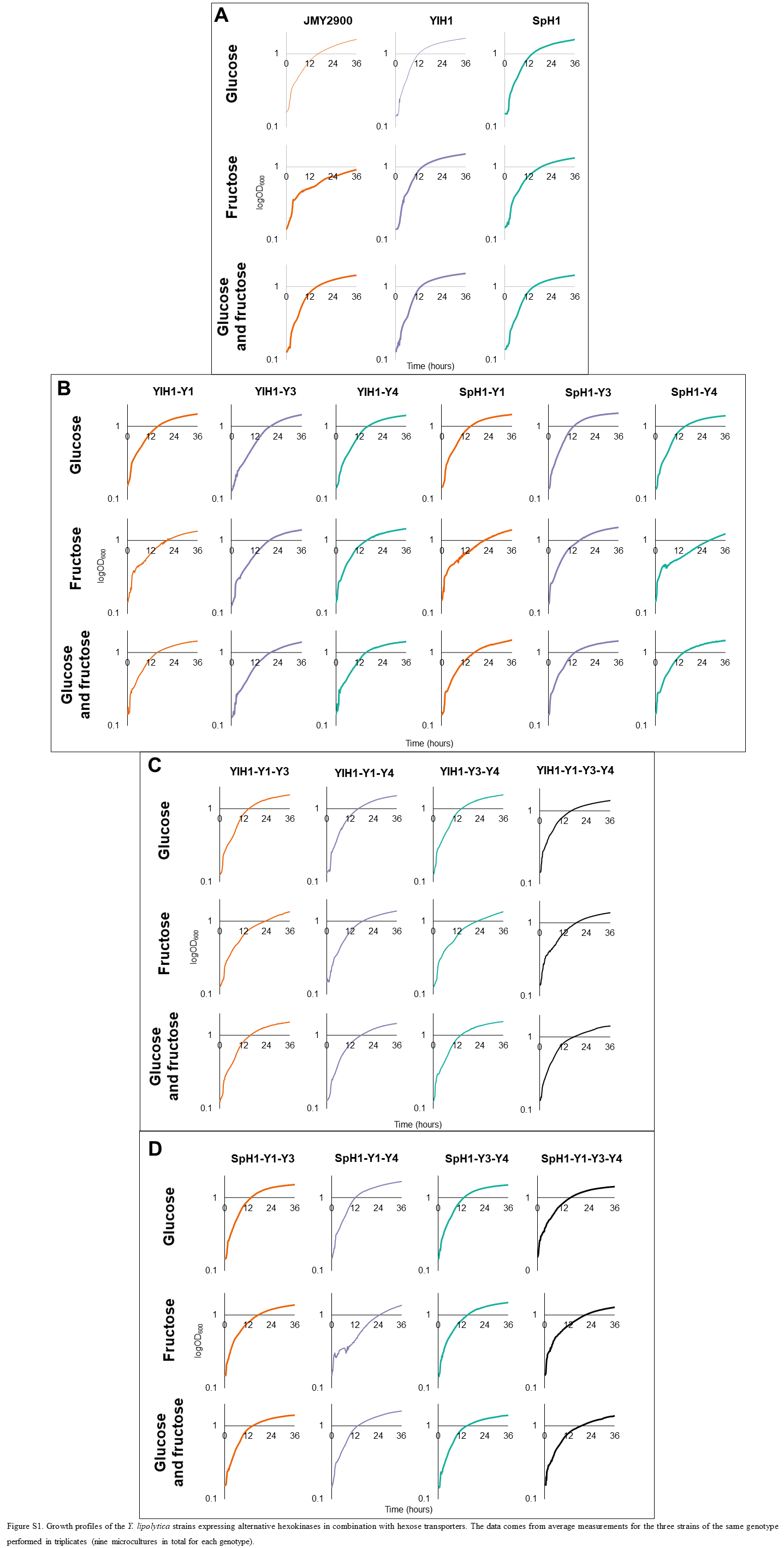

Supplement: Supplementary file 1 [file ijms-22-09282-s001.zip › Figure_S1.tif]

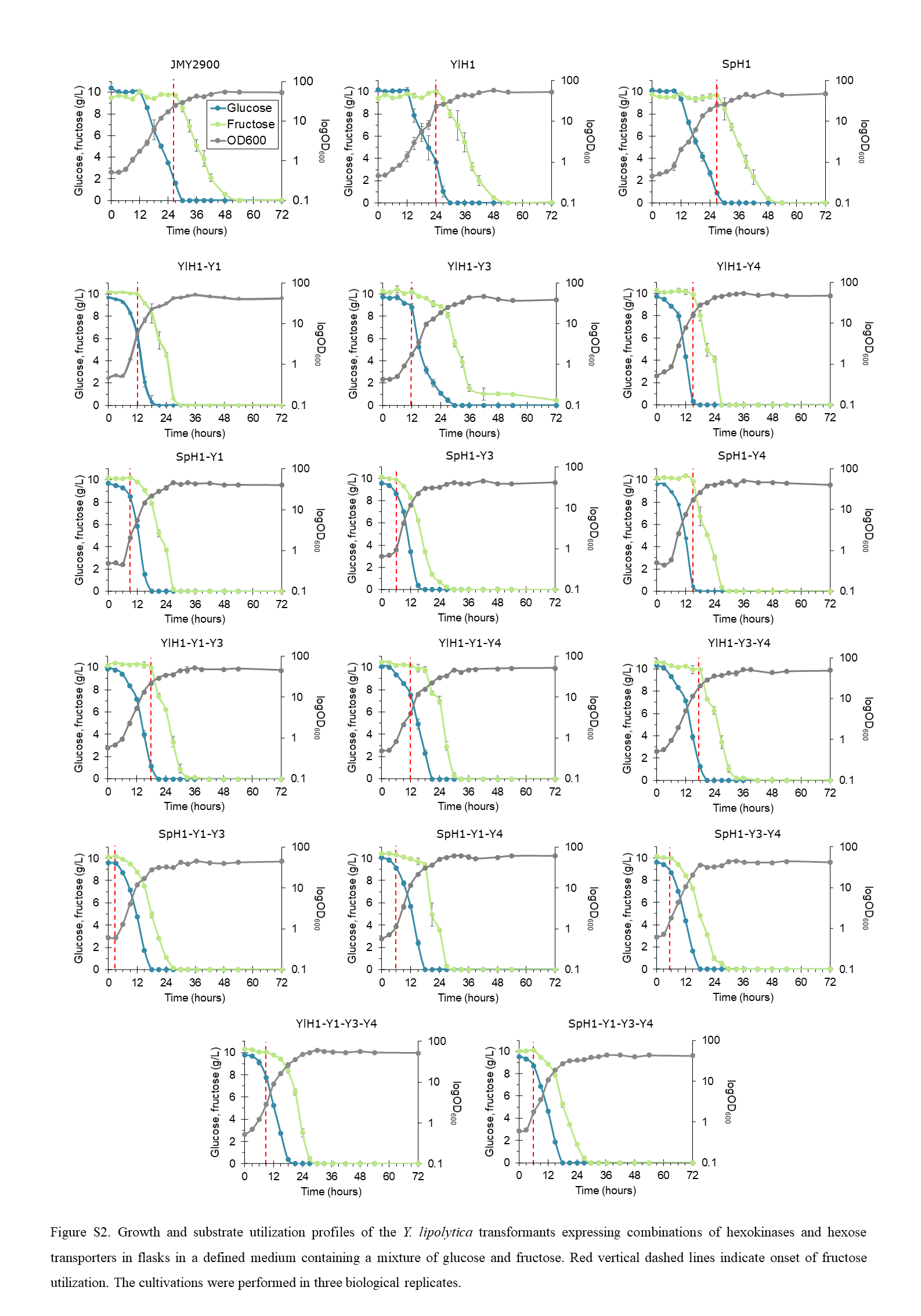

Supplement: Supplementary file 1 [file ijms-22-09282-s001.zip › Figure_S2.tif]

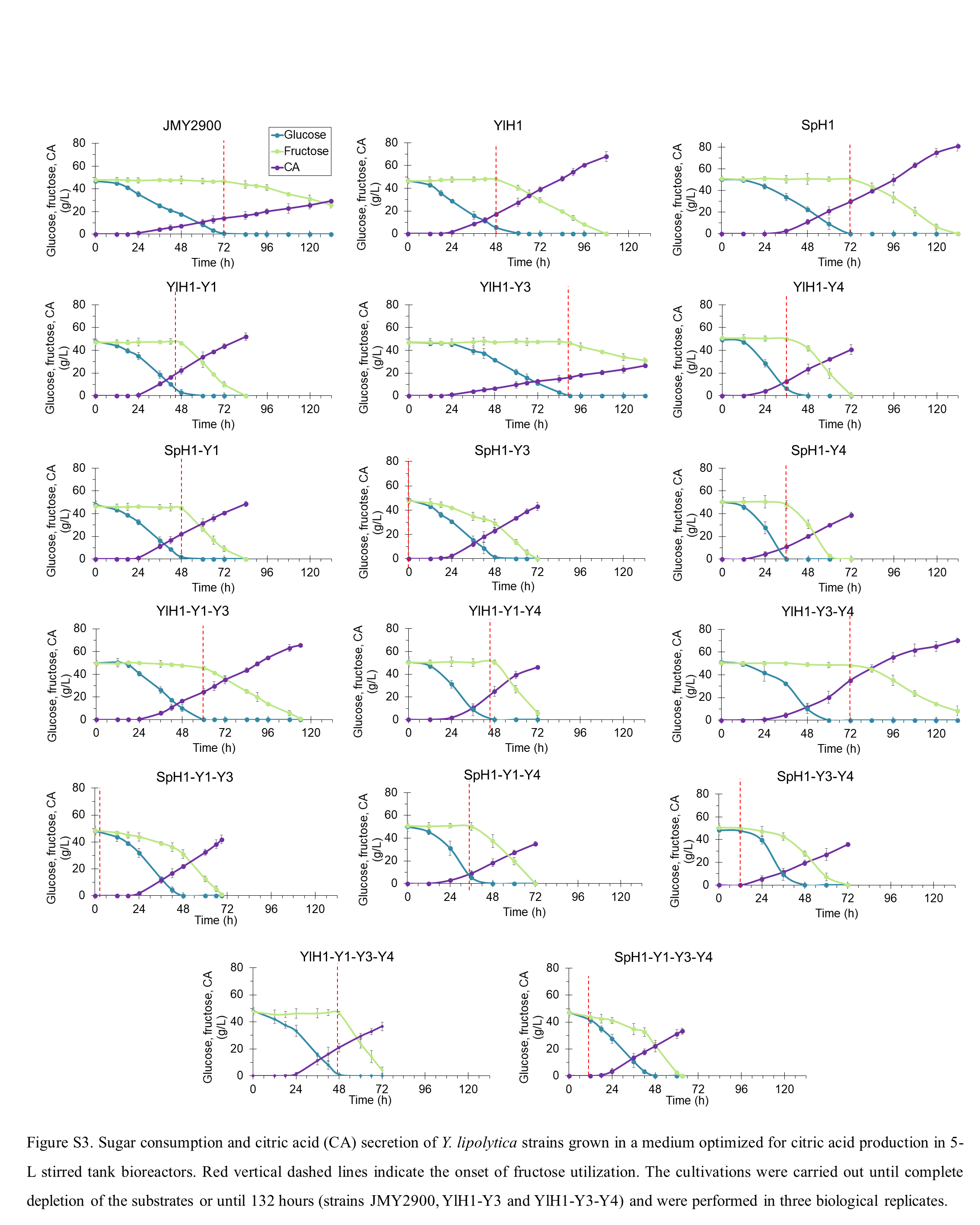

Supplement: Supplementary file 1 [file ijms-22-09282-s001.zip › Figure_S3.tif]

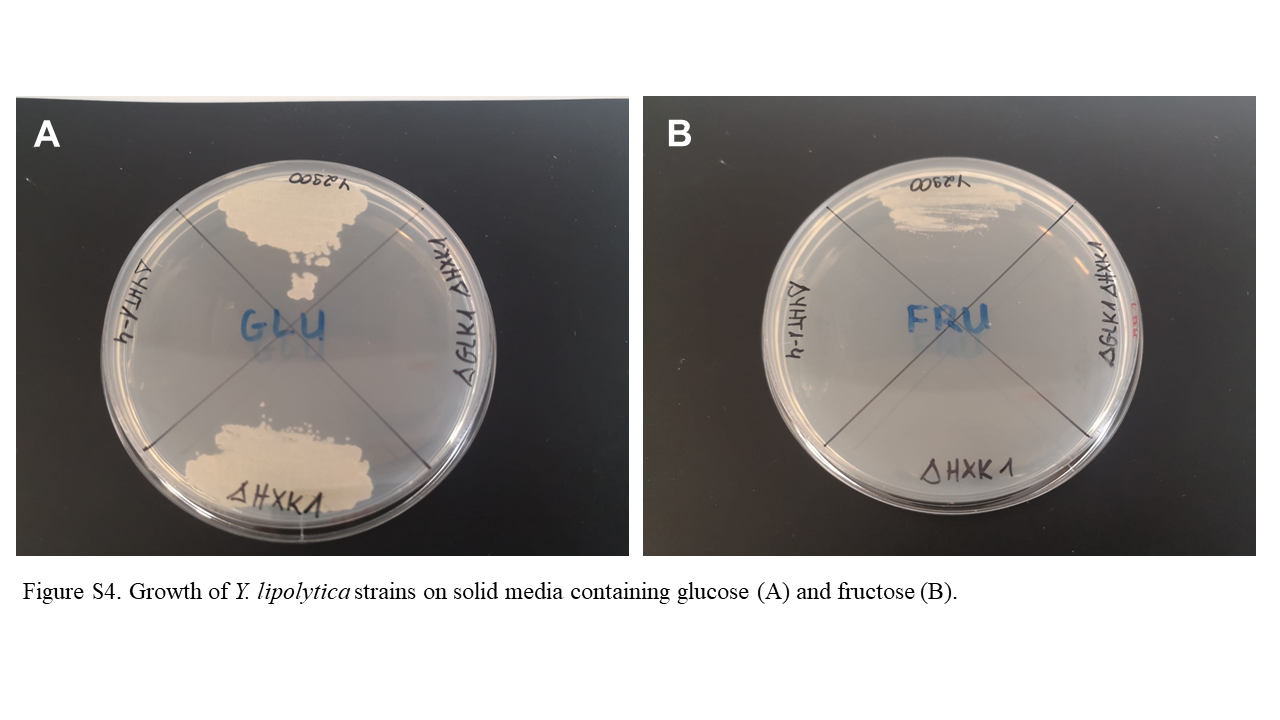

Supplement: Supplementary file 1 [file ijms-22-09282-s001.zip › Figure_S4.tif]

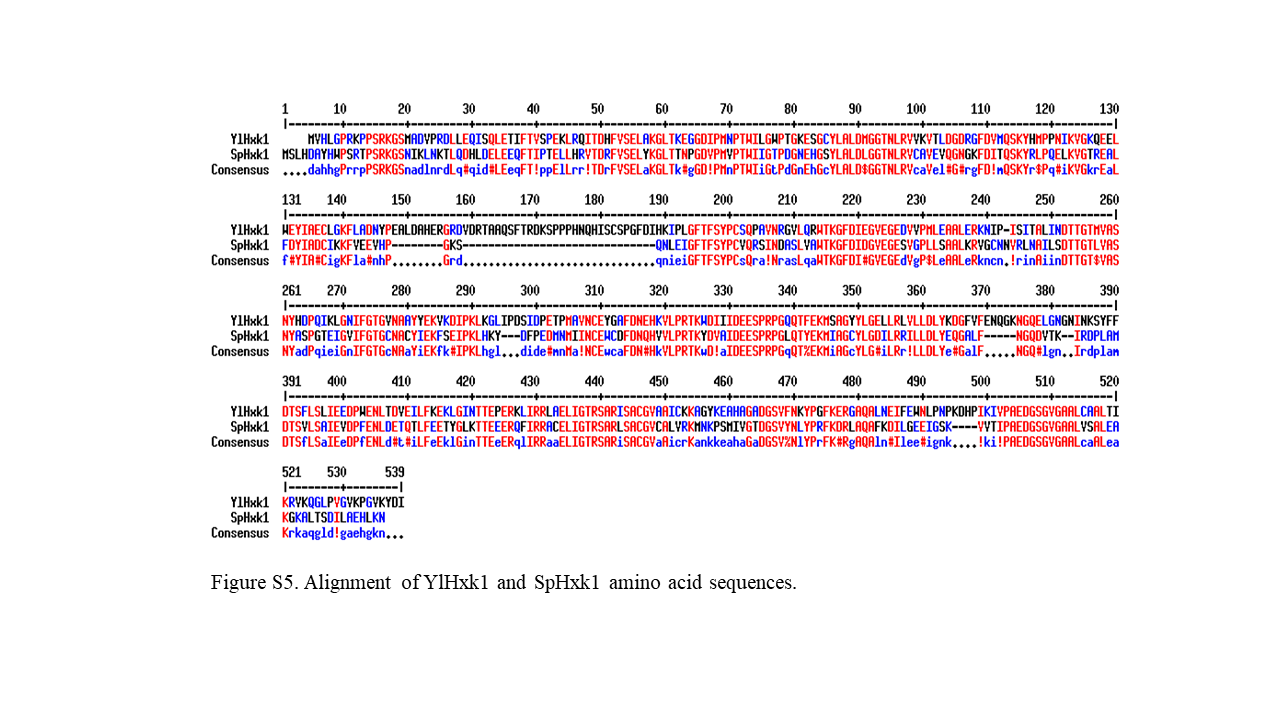

Supplement: Supplementary file 1 [file ijms-22-09282-s001.zip › Figure_S5.tif]

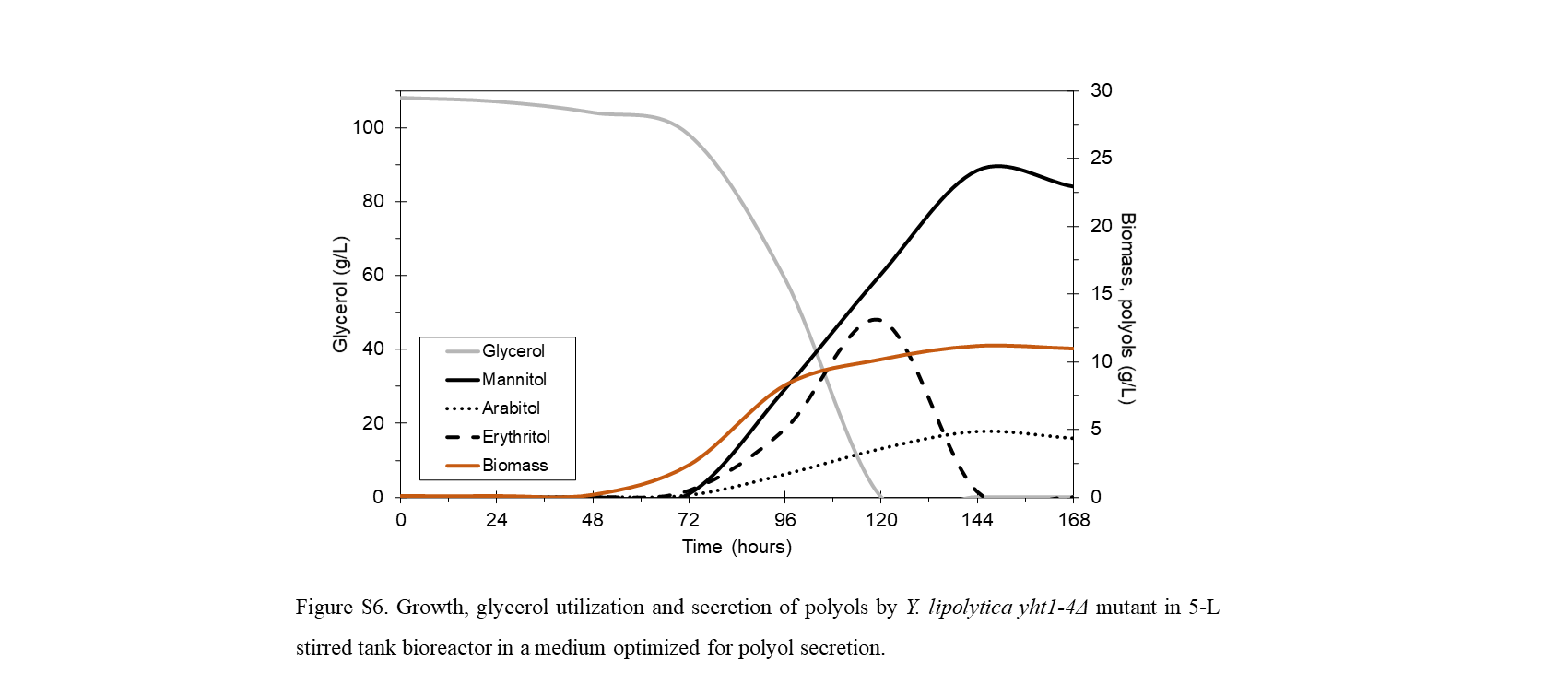

Supplement: Supplementary file 1 [file ijms-22-09282-s001.zip › Figure_S6.tif]

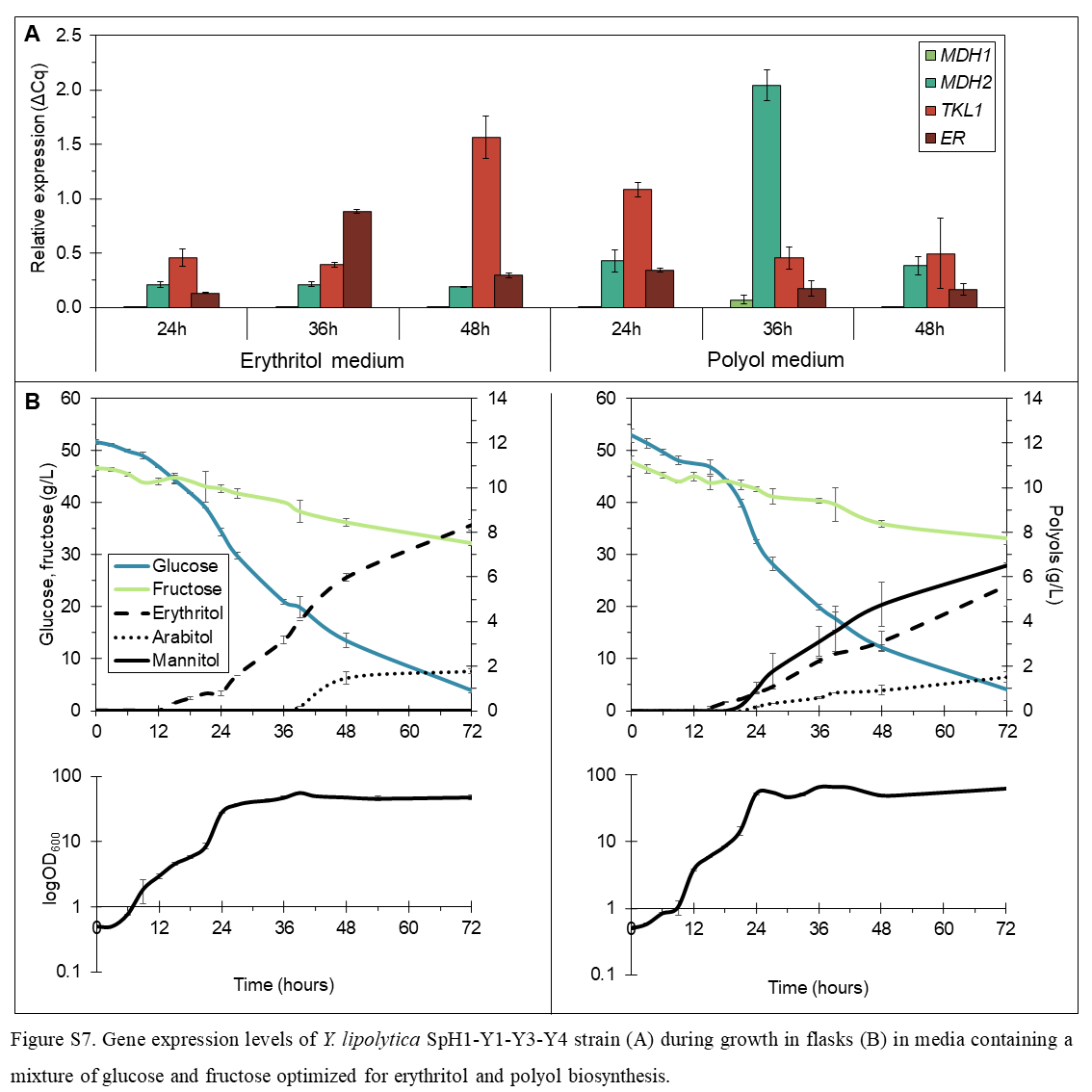

Supplement: Supplementary file 1 [file ijms-22-09282-s001.zip › Figure_S7.tif]
